# A comparison of modeling approaches for static and dynamic prediction of central-line bloodstream infections using electronic health records (part 1): regression models

**DOI:** 10.1186/s41512-025-00199-3

**Published:** 2025-07-21

**Authors:** Shan Gao, Elena Albu, Hein Putter, Pieter Stijnen, Frank E Rademakers, Veerle Cossey, Yves Debaveye, Christel Janssens, Ben Van Calster, Laure Wynants

**Affiliations:** 1 Department of Development and Regeneration, KU Leuven, Herestraat 49 – box 805, Leuven, 3000 Belgium; 2 Department of Biomedical Data Sciences, Leiden University Medical Center, Leiden, the Netherlands; 3 Management Information Reporting Department, University Hospitals Leuven, Leuven, Belgium; 4 Faculty of Medicine, KU Leuven, Leuven, Belgium; 5 Department of Infection Control and Prevention, University Hospitals Leuven, Leuven, Belgium; 6 Department of Cellular and Molecular Medicine, University Hospitals Leuven, Leuven, Belgium; 7 Nursing PICC Team, University Hospitals Leuven, Leuven, Belgium; 8 Leuven Unit for Health Technology Assessment Research (LUHTAR), KU Leuven, Leuven, Belgium; 9 School for Public Health and Primary Care, Maastricht University, Maastricht, the Netherlands

**Keywords:** Risk prediction, Central line–associated bloodstream infection, Dynamic model, Logistic regression, Survival analysis

## Abstract

**Background:**

Hospitals register information in the electronic health records (EHRs) continuously until discharge or death. As such, there is no censoring for in-hospital outcomes. We aimed to compare different static and dynamic regression modeling approaches to predict central line–associated bloodstream infections (CLABSIs) in EHR while accounting for competing events precluding CLABSI.

**Methods:**

We analyzed data from 30,862 catheter episodes at University Hospitals Leuven from 2012 and 2013 to predict 7-day risk of CLABSI. Competing events are discharge and death. Static models using information at catheter onset included logistic, multinomial logistic, Cox, cause-specific hazard, and Fine–Gray regression. Dynamic models updated predictions daily up to 30 days after catheter onset (i.e., landmarks 0 to 30 days) and included landmark supermodel extensions of the static models, separate Fine–Gray models per landmark time, and regularized multi-task learning (RMTL). Model performance was assessed using 100 random 2:1 train-test splits.

**Results:**

The Cox model performed worst of all static models in terms of area under the receiver operating characteristic curve (AUROC) and calibration. Dynamic landmark supermodels reached peak AUROCs between 0.741 and 0.747 at landmark 5. The Cox landmark supermodel had the worst AUROCs (≤ 0.731) and calibration up to landmark 7. Separate Fine–Gray models per landmark performed worst for later landmarks, when the number of patients at risk was low.

**Conclusions:**

Categorical and time-to-event approaches had similar performance in the static and dynamic settings, except Cox models. Ignoring competing risks caused problems for risk prediction in the time-to-event framework (Cox), but not in the categorical framework (logistic regression).

**Supplementary Information:**

The online version contains supplementary material available at 10.1186/s41512-025-00199-3.

## Background

Prediction models play an important role in providing decision support through individualized estimates of disease risk. Electronic health records (EHRs) are commonly used to develop prediction models for in-hospital outcomes. EHR data are complex longitudinal datasets that contain a large number of variables measured at irregular intervals. In time-to-event studies, censoring occurs when the exact time of the prognostic outcome event of interest is unknown for some patients. This can happen due to various reasons, such as the event not occurring within the study’s follow-up period, patients becoming lost to follow-up, or patients withdrawing from the study [1]. In the context of in-hospital outcomes, there is often no censoring in the sense that hospitals register data continuously from the time patients are admitted until they are discharged or deceased, and no patients are lost to follow-up between admission and discharge or death. EHR data are also characterized by the presence of competing events for many prognostic outcomes, i.e., there are competing events that preclude the event of interest from occurring, such as death.

When predicting prognostic outcomes in data without censoring but with competing events, several modeling approaches for categorical and time-to-event outcomes are possible. Operationalizing the outcome as a binary one (experiencing versus not experiencing the event of interest within time *t*) using logistic regression is common [2, 3]. Alternatively, modeling the time to the event of interest using standard Cox proportional hazards regression is often used [2, 3]. Although common, this does not explicitly address competing events and may therefore be expected to perform suboptimally [4, 5]. To account for competing events, time-to-event methods such as cause-specific hazard or Fine–Gray subdistribution hazard models can be used [6]. In the absence of censoring, multinomial regression can also model competing events as outcome categories in addition to the event of interest [7].

For outcomes during admission, there is often an interest in updating predictions over time as the health of the patient changes during their stay. For such dynamic prediction modeling, van Houwelingen described the landmarking approach [8]. Landmarking involves fitting a model that can make or update predictions at a series of time points during follow-up, known as landmarks (LMs), as a function of predictors measured up to the landmark time. This approach is straightforward to implement and computationally simple [9]. An alternative approach is regularized multi-task learning (RMTL), which aims to jointly optimize predictions for multiple related tasks, such as predicting at multiple landmarks [10].

In this study, we aimed to compare various categorical and time-to-event approaches in developing both static and dynamic models, focusing on central line–associated bloodstream infections (CLABSIs). CLABSI, occurring at least 48 h after admission in the absence of infection at another site [11], is a priority for prevention due to its association with prolonged hospital stays, increased healthcare costs, and elevated morbidity and mortality [12–14]. Our analysis utilized EHR data from University Hospitals Leuven (UZ Leuven) to predict the 7-day risk of CLABSI among hospitalized patients with catheters.

## Methods

### Study design and participants

This is a retrospective cohort study of 27,478 patient admissions from the University Hospitals Leuven who were admitted to the hospital and received any type of central venous catheter between January 1, 2012 and December 31, 2013. The study sample included 30,862 patient-catheter episodes. A patient may have multiple catheter episodes if the patient received multiple catheters with more than 48 h in between catheters. Details can be found in Supplementary file 1.

### Study outcome

Our primary focus is on the outcome of CLABSI, which is defined as any laboratory-confirmed bloodstream infection occurring in a patient with a central line or within 48 h after its removal, in accordance with the 2019 definition published by Sciensano, the Scientific Institute of Public Health in Belgium [11]. This definition specifically excludes infections present on admission, secondary infections, skin contamination, and laboratory-confirmed bloodstream infection resulting from mucosal barrier injury. Further details can be found in Supplementary file 1. The outcome to be predicted is CLABSI occurrence within 7 days of the moment of prediction (i.e., at catheter onset or at a later landmark). The time horizon of 7 days was chosen because this was considered the clinically meaningful time interval to intervene according to clinical experts, which included an infection preventionist, an intensivist, and a specialist from the central-line placement team. We discerned two competing events: (1) death or start of palliative care (predictions in palliative care are not actionable due to reduced monitoring intensity) before CLABSI occurrence and (2) discharge from hospital or catheter removal for more than 48 h without CLABSI.

**Fig. 1:**
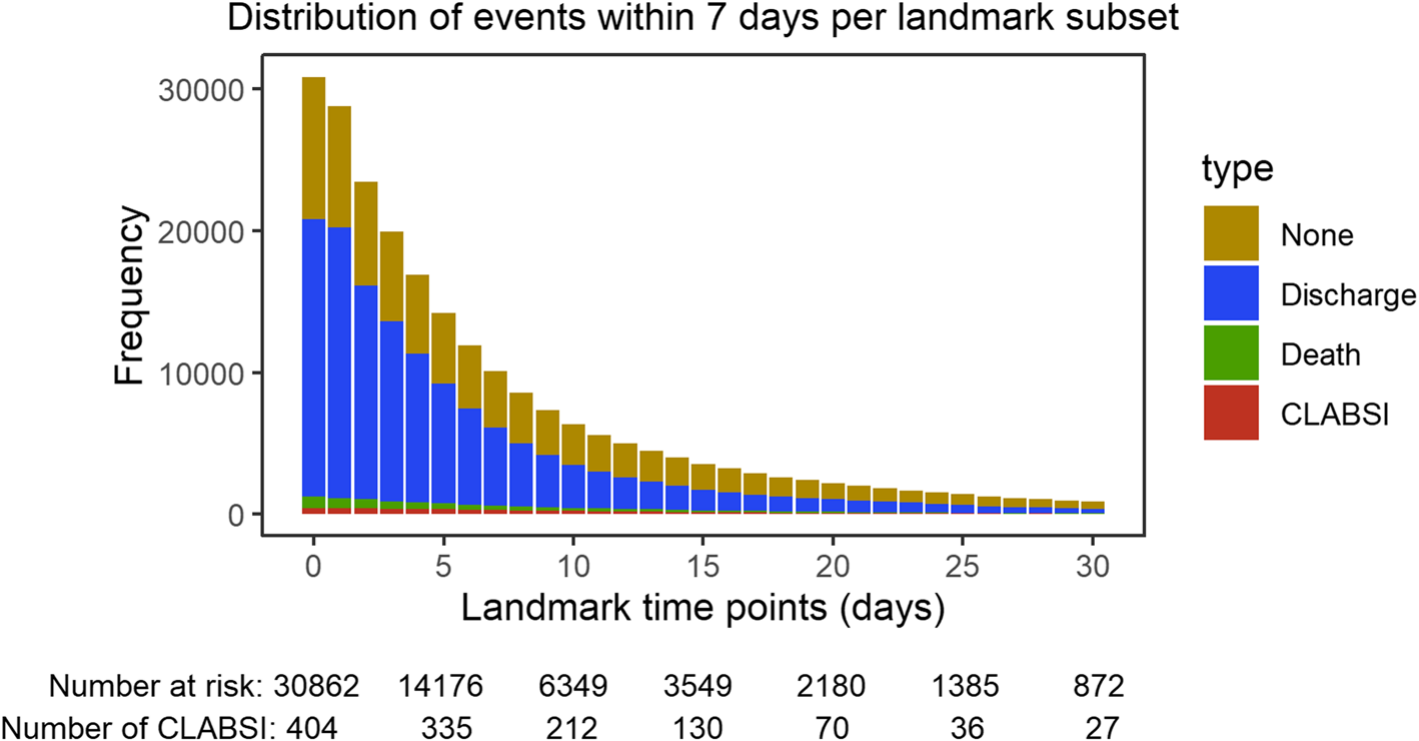
Frequency of outcomes within 7 days for each of the landmark subsets (LM ≤ 30). The total height of the bar is the number at risk

Among 30,862 catheter episodes, 404 resulted in CLABSI within the first 7 days after catheter onset (1.31%). An additional 566 catheter episodes encountered CLABSI beyond the initial 7 days following the onset of catheter episodes (1.83%). Overall, 970 catheter episodes resulted in CLABSI (3.14%) throughout the entire admission. Figure 1 shows the frequencies of the outcomes within 7 days across the catheter episodes at risks in each of the landmark subsets (LM ≤ 30).

### EHR data and predictors

EHR data were extracted from various electronic sources including demographics information, patient admissions, discharges and transitions (e.g., transfers to intensive care units), catheter-related observations, patient medication prescriptions, comorbidities, laboratory tests, and vital signs.

We used 21 predictors (Table 1), which were recorded routinely and chosen based on the domain knowledge collected from the clinical experts and from a systematic review [3]. Twenty of these were time-dependent variables. Individual trajectories of the continuous time-varying variables are illustrated in Supplementary file 3.

**Table 1:** Covariates from the UZ Leuven EHR dataset used in the prediction models

Variable category	Variables	Description	Further information	
Catheter types	Central venous catheter	No (0), yes (1)	In the last 24 h	
	Port-a-cath	No (0), yes (1)	In the last 24 h	
	Tunneled central venous catheter	No (0), yes (1)	In the last 24 h	
	Peripherally inserted central catheter	No (0), yes (1)	In the last 24 h	
Catheter location	Subclavian	No (0), yes (1)	In the last 24 h	
	Jugular	No (0), yes (1)	In the last 24 h	
Medication	Total parenteral nutrition	No (0), yes (1)	In the last 7 days	
	Antibacterials for systematic use	No (0), yes (1)	In the last 7 days	
	Antineoplastic agents	No (0), yes (1)	In the last 7 days	
CLABSI history	History of CLABSI	No (0), yes (1)	In the last 3 months	
Comorbidity	Tumor	No (0), yes (1)	Before current LM	
	Lymphoma	No (0), yes (1)	Before current LM	
	Transplant	No (0), yes (1)	Before current LM	
Physical ward	ICU	No (0), yes (1)	In the last 24 h	
Care modules	Mechanical ventilation	No (0), yes (1)	In the last 24 h	
	Temperature	Unit:$^\circ{\rm C}$	Maximum value in the last 24 h	
	Systolic blood pressure	Unit: mmHg	Last value in the last 24 h	
Laboratory test	WBC count	Unit: 10^9^/L	Last value in the last 24 h	
	CRP	Unit: mg/L	Last value in the last 24 h	
	Positive culture, of any other type than blood	No (0), yes (1)	In the last 17 days	
Admission variable	Whether patients admitted from: home	No (0), yes (1)		

All are timevarying except the admission variable. Due to the collections of various data sources that are at irregular time intervals, we take the dynamic variable values per landmark (24 h). Details can be found in Supplementary file 2

*LM* landmark, *ICU* intensive care unit, *WBC* white blood count, *CRP* C-reactive protein

### Prediction models

#### Static models at the onset of catheter episodes

For static models, we intended to make prediction at the onset of catheter episodes, which occurred either upon catheter placement or upon the registration of the first catheter observation during the admission. Static models at catheter onset included logistic, multinomial logistic, Cox, cause-specific hazard, and Fine–Gray regression. Logistic regression is a straightforward choice for predicting a specific event, providing asymptotically unbiased predictions when the model is correctly specified. Multinomial logistic regression extends this approach by incorporating additional outcome categories, potentially leveraging more information from contrasting both discharged and deceased patients to patients who develop CLABSI instead of combining them in one group. Cox regression is included for illustrative purposes as it is a widely used time-to-event approach. However, it is expected to overestimate the risk of CLABSI and exhibit miscalibration since this approach ignores competing risks and implicitly assumes that CLABSI can also occur after discharge or death. The cause-specific hazard and Fine–Gray regression models further account for competing risks but differ in their handling of risk sets: the cause-specific hazard model treats individuals who have experienced competing events as risk free, while the Fine–Gray approach retains them in the risk set. While these approaches have distinct interpretations of the regression coefficients [15], both can be used to estimate the 7-day CLABSI risk in the presence of competing events [16]. Supplementary file 4 provides a summary of the outcomes and the corresponding formulas for the static models [17–20]. All abovementioned time-to-event models rely on the validity of the proportional hazards assumption. A potential solution to the robustness problem is to apply administrative censoring [21]. All observations are then censored at the target prediction horizon, ensuring that only outcome data directly associated to the survival probability within the specified time window of interest is used [21]. We fitted time-to-event models with and without administrative censoring at the prediction horizon.

#### Dynamic models

For dynamic models, time-varying predictor information is used (Table 1).

The landmark approach in survival analysis involves selecting specific time points, known as"landmarks"{${s}_{0},\dots , {s}_{L}$} at which the risk estimates for an event of interest are updated, using the information on the individuals who survive up to that given landmark time point.

Let *w* be the prediction window of interest. We aimed to create a model to estimate risk at landmark time *s*, knowing an individual’s covariate values at *s*, namely, *Z(s)*, conditioning on being event free at *s*. To create the landmark model, landmark datasets are created for each landmark *s*, using only the data of individuals still at risk at* s*, and applying administrative censoring at *s* + *w* to these individuals. Separate models can be fitted at each landmark *s* for which a prediction is required. However, this is less practical and difficult to communicate with clinical users as a different prediction equation is used for each prediction time point of interest [9]. Alternatively, a landmark supermodel can be fitted after stacking landmark datasets into a super dataset (details can be found in Supplementary file 2).

Landmark supermodels were fitted for binary logistic regression, multinomial logistic regression, Cox survival, cause-specific survival, and Fine–Gray survival models. Supplementary file 4 summarizes the corresponding formulas for predicting CLABSI with dynamic landmark models [22–24]. The concern in developing a landmark supermodel based on the Fine–Gray approach was in constructing landmark datasets that properly accounted for competing events that happened before the landmark in the setting of subdistribution hazard [24]. Liu et al. extended the landmark method to the Fine–Gray model and proposed the landmark proportional subdistribution hazards (PSH) supermodel by transforming each landmark subset into the counting process style before stacking all landmark datasets (details can be found in Supplementary file 2) [25]. Additionally, time-varying inverse probability censored weighting (IPCW) needed to be calculated for the subjects who experienced competing risks [26]. With these necessary changes, the Fine–Gray supermodel stands out from the other supermodels. For comparison, we also constructed separate Fine–Gray models for each landmark.

Lastly, we implemented regularized multi-task learning (RMTL) for simultaneous learning of distinct logistic regression tasks [27]. We defined tasks as landmark specific models, with *t* = 31 tasks in total. Generally, the algorithm uses gradient descent to estimate the task-specific coefficients by minimizing the summation of logistic loss functions across all tasks and two regularization terms, parameterized by ${\lambda }_{1}$ for cross-task regularization (knowledge transfer across tasks), and ${\lambda }_{2}$ for the L2-norm or ridge regularization. For cross-task regularization, we used network-based relatedness to incorporate time-smoothness in the estimated coefficients, which has the effect of shrinking the coefficients of adjacent landmarks toward each other. We tuned ${\lambda }_{1}$ (for knowledge transfer across landmarks) using fivefold cross-validation on the training set and set the ${\lambda }_{2}$ hyperparameter to 0 (no regularization toward zero). The variables in each task train set have been standardized by subtracting the mean of the variable and dividing by the standard deviation. The task test sets have been standardized using the mean and standard deviation of each variable from the corresponding task train set.

### Statistical analysis

#### Model building and validation

**Fig. 2:**
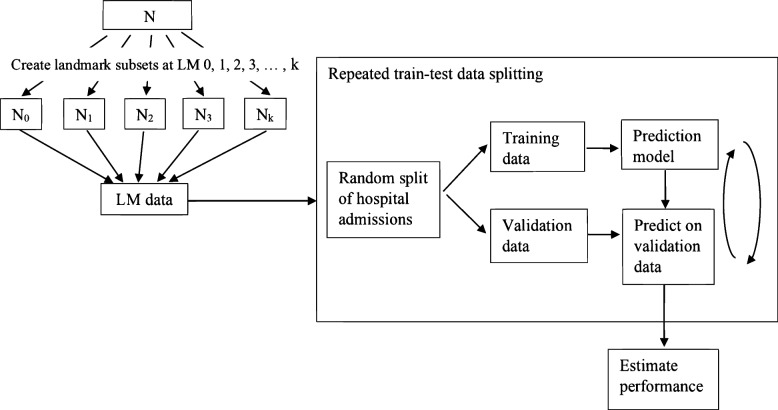
Creation of train-test data for estimating performance

Table 2 lists all models that were fitted. All static and dynamic models used the same 21 predictors, but static models only used predictor values at the onset of catheter episodes. Systolic blood pressure was modeled using restricted cubic splines with three knots. White blood cell count and C-reactive protein were log transformed due to their right-skewed distributions. For dynamic models, smooth baseline hazards were assumed, by including the linear and quadratic landmark time variables over the stacked landmark datasets in model-fitting. In addition, we tested the landmark–covariate interactions for each covariate via the Wald test (α = 0.05). We found that the effects of being on an ICU was significantly dependent on the landmark time (linear and quadratic) and included the interaction terms in our dynamic models to capture the time-dependent effect of this predictor. We used repeated data splitting to develop and validate the models (Fig. 2). The procedure is as follows:

- A random sample of two thirds of hospital admissions from the landmark dataset was used for training; the remaining one third of the admissions were used for model validation. For dynamic models, all landmarks and all catheter episodes of one hospital admission either fell completely in training or test data.
- The candidate models were fitted on the training data.
- The fitted models were used to obtain predicted risk of CLABSI for the test data.
- Model performance measures were evaluated in the test data.
- The steps above were repeated 100 times.

**Table 2:** Summary of statistical methods used for static and dynamic models

Model name	Regression model	Competing risks	Administrative censoring	R package and function
Static				
Cox	Cox	No	No	survival::coxph
Cox-ac	Cox	No	Yes	survival::coxph
CS	Cause specific	Yes	No	riskRegression::CSC
CS-ac	Cause specific	Yes	Yes	riskRegression::CSC
FG	Fine–Gray	Yes	No	riskRegression::FGR
FG-ac	Fine–Gray	Yes	Yes	riskRegression::FGR
LR	Logistic	No	NA	stats::glm
MLR	Multinomial logistic	Yes	NA	nnet:::multinom
Dynamic				
LM Cox	Cox	No	Yes	survival::coxph
LM CS	Cause specific	Yes	Yes	riskRegression::CSC
LM FG	Fine–Gray	Yes	Yes	survival::coxph; survival::finegray
FG-sep	Fine–Gray	Yes	Yes	riskRegression::FGR
LM LR	Logistic	No	NA	stats::glm
LM MLR	Multinomial logistic	Yes	NA	nnet:::multinom
RMTL-ts	RMTL	No	NA	RMTL::MTL

*NA* not applicable

We assessed model performance on test data in terms of discrimination using the area under the receiver operating characteristic curve (AUROC or AUC) [28], calibration (measuring how well the estimated probabilities match the observed probabilities [29]), and overall performance using the scaled Brier score [28] (Table 3). As there was no censoring in the dataset, all performance measures were assessed by treating the outcome as binary. To do so, we evaluated the estimated probability of CLABSI within 7 days against the occurrence of CLABSI within 7 days (yes vs no).

**Table 3:** Assessment of predictive performance

	**Explanation**
Discrimination	
AUROC	Ranges in value from 0 to 1
Calibration	
Calibration slope	Target value is 1. A slope < 1 suggests that estimated risks are too extreme, i.e., too high for patients who are at high risk and too low for patients who are at low risk. A slope > 1 suggests the opposite, i.e., that risk estimates are too moderate [30]
O/E ratio	Target value is 1. Ratio of overall observed outcome proportion to average estimated risk. The O/E ratio < 1 suggests that the model tends to overestimate the risk [31]
ECI	Target value is 0. An averaged squared difference of the predicted probabilities with the estimated observed probabilities [32]. The larger the ECI, the more uncalibrated the model
Overall	
Scaled Brier	Ranges in value from 0 to 1. The percentage reduction in Brier score compared to a null model. The higher the scaled Brier score is, the better the predictions are discriminated and calibrated [33]

*AUROC* area under the receiver operating characteristic curve, *O/E ratio* observed rate/expected rate, *ECI* estimated calibration index

For dynamic models, landmarks beyond day 30 were not considered due to the limited number of catheter episodes (872 catheter episodes, 2.83%) remaining at risk after day 30.

#### Missing value imputation

Missingness percentages of the predictors at all landmarks are shown in Supplementary file 5. One variable (admission source) was imputed with mode value due to its low percentage of missingness (less than 3%). For the remaining variables with missing values, missing data imputations have been performed on each training set using an adaptation of the missForest algorithm for prediction settings [34]. The missing values were first imputed with mean/mode and then iteratively imputed using random forest models for 5 iterations. Using a model-based imputation method is commonly expected to reduce uncertainty and missing data bias compared to complete case analysis and mean/mode imputation. In particular, applying a random forest–based imputation helps preserve interactions and nonlinear relations between variables [35]. The outcome was not included in the imputation model to reflect the real-world prediction setting, where the outcome is unknown at the time of prediction. Test datasets were imputed using the missForestPredict imputation models learned on the matching training dataset to mimic prediction model use at the bedside [36].

#### Sample size and software

The sample size calculation is detailed in Supplementary file 6. Sample size was calculated using the *pmsampsize* package [37]. All analyses were performed using R v4.3.2. Software packages used for building static and dynamic models are shown also in Table 2. Details regarding the R code for fitting and evaluating the models can be accessed via Supplementary file 7. The codes were illustrated using the UZ Leuven EHR data. As it is not permitted to share the original data, a manually created example dataset is shared in Supplementary file 2 with sensitive information being replaced.

### Ethics

The study adhered to the principles of the Declaration of Helsinki (current version), the principles of Good Clinical Practice (GCP), and all relevant regulatory requirements. Ethical review was sought from the Ethics Committee Research UZ/KU Leuven, Belgium, which is the local ethics committee at UZ Leuven (https://admin.kuleuven.be/raden/en/ethics-committee-research-uz-kuleuven#). The collection, processing, and disclosure of personal data, such as patient health and medical information, were in compliance with applicable personal data protection and the processing of personal data (Directive 95/46/EC and Belgian law of December 8, 1992 on the Protection of the Privacy in relation to the Processing of Personal Data). Patient stay identifiers were coded using the pseudo-identifier available in the data warehouse of the participating hospital.

## Results

### Static models at the onset of catheter episodes

Descriptive statistics of variables at the onset of the catheter episode are provided in Supplementary file 8. All models showed similar discrimination and calibration performances except Cox proportional hazard models (Table 4 and Supplementary file 9). Cox models had lower AUROC values and clear miscalibration compared to the other models. The cause-specific model without administrative censoring had the highest AUROC (median 0.721). In terms of calibration, the cause-specific and Fine–Gray model with administrative censoring, as well as binary and multinomial logistic models showed well-calibrated results. For overall performance, cause-specific models, either with or without administrative censoring, as well as multinomial logistic model showed higher scaled Brier scores than the other models. Coefficient estimates of all static models are provided in Supplementary file 10. Additionally, we evaluated the performance of all static models with the inclusion of age as a predictor (Supplementary file 11), but observed no substantial improvements.

**Table 4:** Summary of the performance measures (mean with 95% CI) for static models

**Model**	**AUROC**	**Calibration** **slope**	**O/E ratio**	**ECI**	**Scaled Brier**
Cox-ac	0.649 (0.644, 0.654)	1.083 (1.052, 1.115)	0.603 (0.592, 0.615)	0.017 (0.016, 0.019)	− 0.001 (− 0.002, − 0.000)
Cox	0.656 (0.650, 0.662)	1.416 (1.373, 1.459)	0.583 (0.572, 0.593)	0.014 (0.013, 0.014)	0.001 (− 0.000, 0.001)
CS-ac	0.715 (0.711, 0.718)	0.940 (0.920, 0.960)	1.028 (1.009, 1.047)	0.005 (0.004, 0.005)	0.008 (0.008, 0.009)
CS	0.721 (0.718, 0.725)	1.204 (1.180, 1.228)	1.052 (1.033, 1.071)	0.003 (0.002, 0.003)	0.009 (0.009, 0.010)
FG-ac	0.705 (0.702, 0.709)	0.913 (0.893, 0.932)	1.011 (0.992, 1.030)	0.005 (0.005, 0.006)	0.007 (0.006, 0.008)
FG	0.718 (0.715, 0.722)	0.870 (0.854, 0.885)	1.014 (0.995, 1.032)	0.005 (0.005, 0.006)	0.005 (0.004, 0.006)
LR	0.706 (0.702, 0.709)	0.909 (0.890, 0.929)	1.009 (0.990, 1.028)	0.005 (0.005, 0.006)	0.007 (0.006, 0.008)
MLR	0.713 (0.710, 0.717)	0.938 (0.917, 0.958)	1.009 (0.990, 1.028)	0.004 (0.004, 0.005)	0.009 (0.008, 0.009)

### Dynamic models

**Fig. 3:**
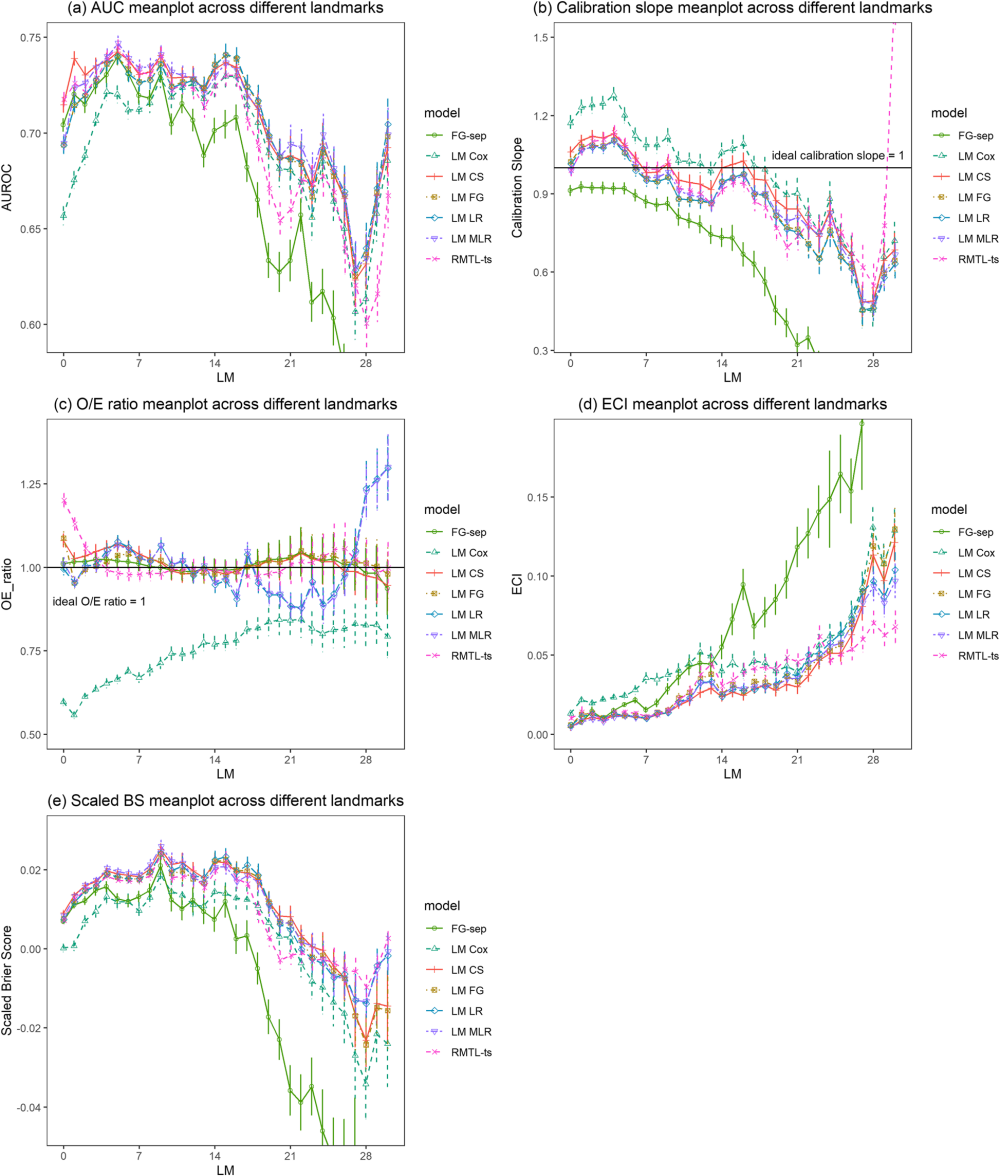
Comparison of performance metrics of dynamic models across landmarks. The vertical Y-axis was truncated for clarity. Minimum mean observed AUROC was 0.535, minimum/maximum mean observed calibration slope was 0.093/1.742, maximum mean observed ECI was 0.386, minimum mean observed scaled BS was −0.133

Generally, discrimination increased from landmark day 0 to landmark day 5, then slightly decreased up to landmark day 16, and decreased strongly thereafter (Fig. 3 and Supplementary file 12). It can be noticed that the performance metrics of separate Fine–Gray landmark model started to diverge from others since landmark day 14, coinciding with the emergence of convergence issues from landmark day 14 onward (details can be found in Supplementary file 13). The Cox landmark supermodel had clearly lower AUROC than all other models up to landmark 7. The landmark cause-specific supermodel had the highest AUROC (mean AUROC up to 0.739) for predictions up to 3 days after the onset of catheter episodes. For predictions between 4 and 13 days after placement, the landmark multinomial logistic model had a slightly higher AUROC than other models (mean AUROC up to 0.747). After landmark day 13, the landmark logistic and Fine–Gray model had higher AUROC values than the other models until landmark day 20. The approach based on separate Fine–Gray models per landmark had worse AUROCs than other models from landmark 10 onwards.

The Cox landmark supermodel was less competitive in terms of calibration compared to other dynamic models. The other models demonstrated comparable performance. For binary and multinomial logistic landmark supermodels, calibration deteriorated after landmark day 14 in terms of O/E ratio. In contrast, the Fine–Gray and cause-specific landmark supermodels maintained stable O/E ratios. For all supermodels and RMTL, calibration slopes were above 1 before landmark day 7, implying that risk estimates tended to be too close to the average CLABSI risk. For separate Fine–Gray landmark models, calibration slopes were always below 1, indicating overfitting.

In terms of scaled Brier score, the multinomial logistic landmark supermodel was superior to the other models before landmark day 11. Afterwards, the binary logistic landmark supermodel showed a slight advantage until landmark day 18.

Coefficient estimates of all dynamic models are provided in Supplementary file 10. Supplementary file 14 presents the distribution of predicted risks from the dynamic models across landmark time points. In addition, the inclusion of age in the dynamic models (Supplementary file 11) did not yield notable improvements in predictive performance.

## Discussion

In this study, we compared different statistical methods for predicting the risk of CLABSI within 7 days during hospital admission using EHR data in which there was no censoring. Both static and dynamic models were fitted and compared. Discrimination, calibration, and overall predictive performance showed relatively minor distinctions between the models, except that the Cox proportional hazards models performed noticeably worse than the other models. This was expected as the Cox model assumes that the competing risks do not exist (i.e., patients cannot be discharged and cannot die) and therefore overestimates the risk of CLABSI [6]. Logistic regression models performed very well, as competing events were not censored (and assumed to have the same CLABSI risk as those still in the risk set) but counted as non-events. When using separate Fine–Gray models at each landmark, performance was worse than landmark competing risk supermodels from early landmarks onwards [25, 26]. This might be attributed to the substantial decrease in sample size at later landmarks, leading to overfitting, in combination with the fact that separate models do not borrow information from adjacent landmarks. Multinomial regression with competing events as separate outcome classes showed a small advantage over binary logistic regression, possibly due to its ability to preserve essential information concerning the different outcome categories. The RMTL model performed better than the Cox and separate Fine–Gray landmark models, but did not perform better than the other models. For dynamic models, discrimination and calibration performance varied across landmarks.

Based on our results, for prognostic prediction without censoring, logistic modeling can be a good choice. In our setting, accounting for competing events through multinomial logistic modeling was not necessary. When time-to-event models are used, it is crucial to account for competing events to avoid strong miscalibration, as highlighted by the poorer performance of the standard Cox models compared to the cause-specific and Fine–Gray models. Furthermore, cause-specific time-to-event models showed slightly better predictive performance than Fine–Gray models, aligning with recent research findings [16]. These findings are useful to inform and improve the development of dynamic models in electronic health records. For example, in a recent systematic review covering 16 prediction models for CLABSI, 13 models used a binary outcome (9 with logistic regression, 2 with XGBoost, 1 with random forests, and 1 with naïve Bayes) and 3 modeled time to CLABSI using Cox proportional hazards regression [3]. Researchers should take note of the potential overestimation and loss of discrimination associated with the Cox model observed in the current study and previous research [6]. The review did not identify any time-to-event model that included competing events. Only one model used a fixed prediction horizon [38], the other 15 models considered CLABSI at any time during admission. Even though logistic regression can be a valid option in this specific setting, the lack of a fixed prediction horizon leads to poorly interpretable and usable models. Only one model in the systematic review attempted dynamic prediction; the other 15 models focused on static prediction at the moment of catheter onset. Nonetheless, dynamic prediction has been recognized as a straightforward and useful approach to in-hospital prediction [39] and other longitudinal prediction problems where predictions should be updated over time in response to changing patient data. As both intercepts and covariate effects may shift over time and lead to inaccurate risk estimates, naively relying on a static model throughout a patient’s hospitalization is problematic. This is illustrated by the changing performance of dynamic models across landmarks, and of the separate Fine–Gray models at landmarks 0 to 5, before its performance started to plummet. Several dynamic modeling approaches are available [40]. In our current paper, we focused on landmarking and RMTL. Joint models may have a slightly better predictive performance if they are correctly specified, but are more computationally demanding, making them less suitable for EHR data where large numbers of predictors change. We have given practical recommendations to improve the data preparation and clinical applicability for dynamic prediction in hospitals elsewhere [41].

Landmark supermodels are valuable tools for dynamic prognostic modeling. It is worth noticing that in this study we use the landmark approach in the competing risk setting, applying it to the cause-specific and Fine–Gray model. Though there have been studies that applied a landmark approach to the Cox proportional hazards model [42, 43], there are few studies about its application in competing risks survival models. Nicolaie et al. extended the landmark model for ordinary survival data to address the problem of dynamic prediction in the presence of competing risks, based on the cause-specific hazards and dynamic pseudo-observation, respectively [24, 44]. In this study, we fitted the landmark supermodel to the stacked dataset across all landmarks by smoothing baseline hazards [9]. However, the calibration performance requires discussion. The calibration slopes were above 1 at early landmarks and below 1 at later landmarks (Fig. 3b). Calibration plots for the landmark cause-specific supermodel per landmark (Supplementary file 15) suggest that the calibration slopes are the result of underestimation of risks between 0.02 and 0.07 at lower landmarks and overestimation of risks above 0.05 at later landmarks. Improving dynamic calibration in practice requires further research. The obtained result may suggest that dynamic shrinkage approaches could help, or dedicated approaches to select interactions between predictors and landmark. We used a simple approach to select interactions, resulting in the inclusion of the interaction between landmark and only one predictor. Perhaps better selection methods should be identified or sample size considerations should attach more importance to the potential variation of predictor coefficients across landmarks.

Our study also has limitations. First, the predictors used in this study were selected based on insights from a previous systematic review and domain knowledge from clinical experts at UZ Leuven, who rated the importance of predictors for CLABSI risk. These predictors were chosen specifically for CLABSI prediction, without explicitly including predictive variables for other competing events such as death and discharge. Although the prediction of the competing risks is expected to improve by including predictors of the competing events, the prediction of the event of interest might deteriorate in practice if the predictor set becomes too large for the available sample size. Further research should explore whether including strong predictors for all type of events, or applying different sets of variables for competing events, can improve model performance. Second, our models did not fully capture variable dynamics by incorporating the full history of dynamic predictors within a catheter episode. We explored models that included two lagged values and differences between consecutive landmark values but found no improvement in predictive performance. Therefore, for the current methodological comparison, we opted to present the simplest version while maintaining a focus on evaluating the impact of different modeling choices. Third, the study's reliance on data from a single hospital for the years 2012 to 2013 limits the generalizability of its findings, as patient demographics and healthcare practices can vary significantly across different settings. Fourth, the choice of a 7-day prediction horizon was based on clinical relevance within the UZ Leuven context. Alternative timeframes may be more appropriate in other settings depending on local care practices and patient characteristics. In addition, given the potential delay between CLABSI onset and its detection, further research should explore how the choice of prediction horizon impacts on model performance and clinical utility. For example, do models mainly identify CLABSI early in the prediction horizon, or are models also able to identify late infections or even infections beyond the adopted prediction horizon of 7 days? We encourage other hospitals interested in this topic to use the codes provided in Supplementary file 7 to determine the most suitable modeling approach for their specific hospital environments.

We focused on regression models because methodology for dynamic prediction is well established and because regression models were most commonly used in existing CLABSI prediction models [3]. In a subsequent accompanying paper, we investigated the use of random forest algorithms in the same context because such machine learning approaches may automatically detect and include nonlinear and nonadditive associations between predictors and outcome as well as interactions with landmark time [45]. The results of the accompanying paper suggest that random forest models had better discrimination and calibration compared to the regression models.

While the ultimate goal of developing a valid dynamic risk prediction model is to help enhance clinical decision-making and patient outcomes, this study represents an initial step by comparing different modeling approaches in both static and dynamic settings. If models can predict the 7-day risk of CLABSI sufficiently well, they can support clinicians to timely intervene with targeted preventive measures. For instance, early identification of high-risk patients can prompt intensified monitoring, timely catheter removal when clinically indicated, and tailored infection prevention strategies. The dynamic regression modeling approaches studied here offer a robust framework that continuously updates risk assessments, ensuring close monitoring throughout patients’ hospital stay, thereby improving overall patient safety and healthcare efficiency.

## Conclusions

In conclusion, our study compared various statistical approaches for predicting CLABSI within 7 days during hospital admission using EHR data without censoring from UZ Leuven. In the absence of censoring, time-to-event, logistic, and multinomial regression models yielded comparable predictive performance in static and dynamic prediction. However, Cox models, which overlooked competing events that may preclude the occurrence of the outcome of interest, exhibited inferior performance. Our study applied the landmark approach to competing risk settings, highlighting the importance of considering competing events in predictive modeling. Overall, our findings underscored the significance of appropriate model selection and the consideration of competing risks for accurate risk prediction in clinical settings.

## Supplementary Information

Additional file 1: Supplementary materials.

## Data Availability

The data underlying this article cannot be shared publicly due to for the privacy of individuals that participated in the study. Data are located in controlled access data storage at UZ Leuven.
