# Supplementary material for: A comparison of modeling approaches for static and dynamic prediction of central-line bloodstream infections using electronic health records (part 1): regression models
Source: Diagn Progn Res. 2025 Jul 21;9:20. doi: 10.1186/s41512-025-00199-3 (PMC12278581; doi:10.1186/s41512-025-00199-3)
Supplement: Supplementary file 1 — Additional file 1: Supplementary materials. [file 41512_2025_199_MOESM1_ESM.pdf]

Supplementary file 1: CLABSI in UZ LEUVEN

The retrospective cohort study consists of patients from the University Hospitals Leuven who were admitted to the hospital and received a catheter between January 2012 and December 2013.

- **Inclusion criteria**

Only hospital stays in participating hospitals with admission starting from January 1st 2012 up to December 30th 2013 were analyzed. Only patients who had a catheter of the following types were included, in accordance with the definitions of CLABSI of the Hospital Hygiene Department:

- Deep venous catheter
- Peripherally inserted central catheter (PICC) (open and valve)
- Midline catheter (open and valve)
- Tunneled dialysis catheter
- Non-tunneled dialysis catheter
- Umbilical catheter
- Port-a-cath
- Hickman catheter

Excluded were: rapid infusion system (RIS) catheters, Swan-Ganz catheters, Coolgard catheter introducers, pacemakers, arterial catheter, peripheral venous catheter, Extracorporeal membrane oxygenation (ECMO), intra-aortic balloon pump (IABP) Patient-controlled epidural analgesia (PCEA), Pulse index Contour Continuous Cardiac Output (PICCO) .

Patients that had only a dialysis catheter were included only if they have an ICU admission between January 2012 and December 2013, due to the data extraction constraint.

- **Exclusion criteria**

Patients in the neonatology department were documented using a paper-based workflow before October 2013 and did not have electronic records in the system. Thus hospital admissions for patients under the age of 12 weeks have been excluded from the analysis.

- **Catheter episodes**

Considering that there were situations that patients received more than one catheter simultaneously or consecutively in ICU or in other hospital wards, it is difficult to distinguish the effect of those catheters on the risk of CLABSI. Thus, a scheme was developed to make a difference between these situations.

- When the patient received only one catheter, this was regarded as one observation. Its time at risk is the time interval from the catheter placement to the catheter removal plus 48 hours, according to the definition of CLABSI [1].

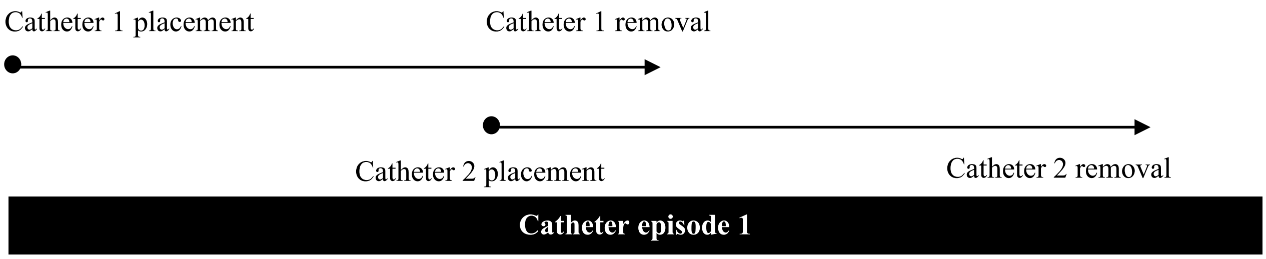

- If the same patient received two catheters and the time interval between these two catheters was less than 48 hours, we treat them as one observation, which means that their time at risk are counted together, from the start of the earlier catheter to the end of the later catheter.

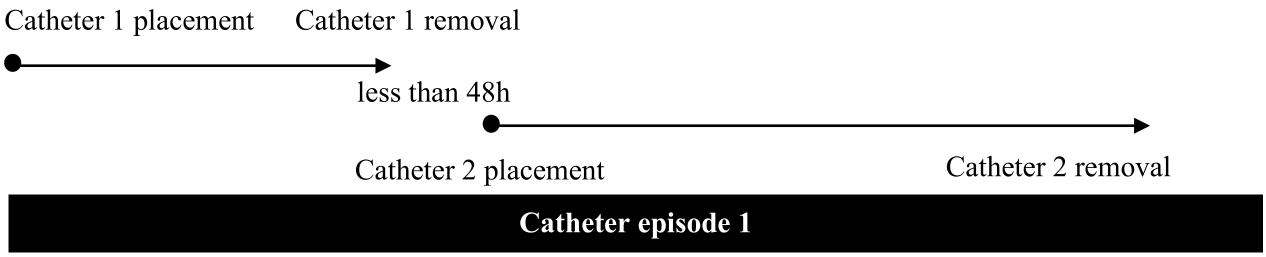

- If the same patients received two catheters and the time interval between these two catheters was more than 48 hours, we treat them as two observations, that is, their time at risk are counted separately.

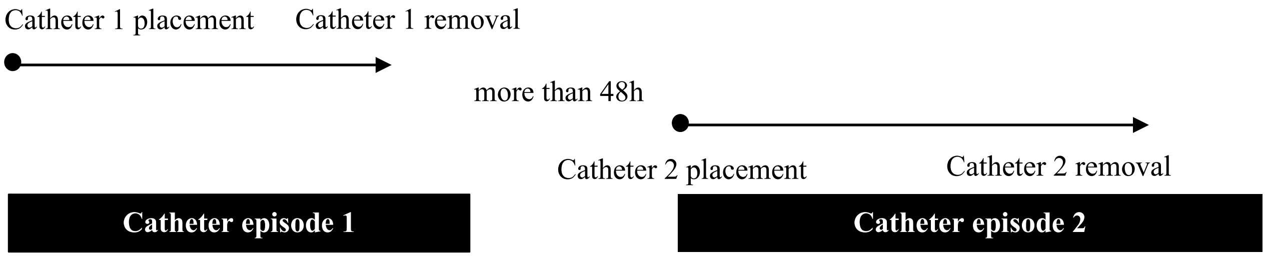

- **Outcome**

There are three types of events (CLABSI, death and discharge) considered in this analysis.

- CLABSI: any laboratory-confirmed bloodstream infection (LCBSI) for a patient with central line or within 48 hours after the central line removal. The CLABSI definition follows the Sciensano definition published in 2019 [2], with specific criteria excluding infection present on admission, secondary infections, skin contamination, and mucosal barrier injury LCBSI. Symptoms criteria are not checked, and it is assumed that cultures are ordered based on relevant symptoms. The time window for secondary infections is not clearly defined in Sciensano, and we use the time window of the 17 days, considering the CLABSI episode length of 14 days plus the time window of 3 days.
- Discharge: hospital discharge or 48 hours after catheter removal, whichever happens first. A patient is still considered at risk within 48 hours after catheter removal.
- Death: Either the first contact with palliative care during admission, transfer to palliative care or patient death, whichever happens first. Patients are not closely monitored in palliative care and predictions for this ward are deemed non-actionable, thus of limited value since there are minimal opportunities to prevent CLABSI events in this context.

Supplementary file 2: stacked dataset

The landmarking approach for dynamic prediction of survival was initially described by van Houwelingen [1]. In brief, at a given landmark time s where a prediction is to be made, the data are restricted to individuals who have not yet experienced the event. Predictor values available up to the landmark time are used as covariates in a model for the probability of survival up to some time horizon, conditional on survival to the landmark time. Typically, the focus is on survival to a single time horizon w, and censoring is imposed at w so that only events up to that time $s+w$ are used in the survival analysis. In this approach, the dataset is transformed into multiple censored datasets based on predefined s and w. Traditionally, a separate cox proportional hazards model can be applied to each landmark dataset and predictions can then be made at each landmark time point. Moreover, a supermodel can be fitted on the stacked super dataset and the landmark supermodel may combine these models by introducing smoothing to permit risk prediction at any landmark. Then dynamic risk prediction can be performed by using the most up-to-date value of patients' covariate values.

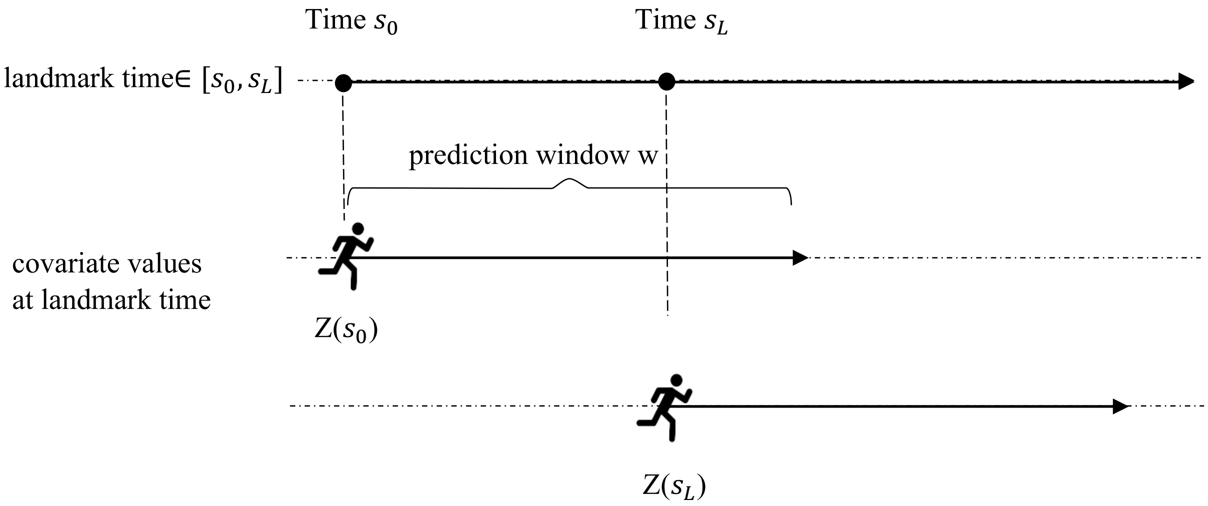

To fit a landmark Cox supermodel [3,4], a stacked dataset is constructed by: (i) firstly selecting a set of landmark points s from $[s_0, s_L]$; (ii) then creating a landmark subset by selecting the subjects who have not yet failed from any cause at s and adding an administrative censoring at the prediction horizon $s+w$; (iii) finally stacking all the individual landmark subsets into a super prediction dataset.

The following table is an example of the stacked super dataset (with pseudo-anonymization) which is used to develop the dynamic models. The original data is not allowed to share, thus sensitive information are replaced with manually created synthetic data in the following table.

Table S1: example data for landmark supermodels (except Fine-Gray supermodel)

ID	LM	eventtime	type[a]	ICU unit	CRP
**1**	0	4.42	1	0	28.6
**1**	1	4.42	1	0	50.7
**1**	2	4.42	1	0	46.2
**1**	3	4.42	1	1	46.7
**1**	4	4.42	1	0	21.2
**2**	0	7.00[b]	0	1	86.1
**2**	1	8.00[b]	0	1	99.7
**2**	2	9.00[b]	0	1	87.5
**2**	3	9.34	1	1	51.2
**2**	4	9.34	1	1	40.7
**2**	5	9.34	1	1	27.5
**2**	6	9.34	1	1	19.6
**2**	7	9.34	1	1	29.3
**2**	8	9.34	1	1	17.8
**2**	9	9.34	1	1	9.6
**3**	0	1.29	2	1	90.1
**3**	1	1.29	2	1	131.4
**4**	0	4.56	3	0	41.1
**4**	1	4.56	3	0	157.3
**4**	2	4.56	3	0	167.9
**4**	3	4.56	3	0	134.5
**4**	4	4.56	3	0	41.6

LM: landmark time; eventtime: time when any type of event happened; type: type of the event; ICU unit: binary indicator, whether the patient now (at the exact second of the current LM) is in ICU; CRP: continuous variable, last value of C-reactive protein test since previous LM. Unit: mg/L.

[a]type=1 (CLABSI); type=2 (Death); type=3 (Discharge); type=0 (Censored)

[b]Discharge here means catheter removal. As prediction time window is 7-day from each landmark time, individuals who are free of event up to 7 days of follow-up from the corresponding LM are administratively censored.

For landmark super subdistribution models, we used another expanded example dataset here for explanation. The standard estimator of the cause-specific cumulative incidence function can be written as a Kaplan-Meier type product-limit estimator with the estimator $d_j(t_i)/r(t_i)$ replaced by an estimator of the subdistribution hazard ($r(t_i)$ is the observed number at risk). This estimator of the subdistribution hazard $\widehat{\lambda_j}(t_i)$has the form $(d_j(t_i)/r^*(t_i))$ with $r^*(t_i)$ obtained by reweighting individuals who had a competing event before t_i. Thus, by creating a data set where individuals with an earlier competing event still contribute to the risk set with a weight, we can estimate the subdistribution hazard using the standard counting process approach. The following example is an expanded dataset created based on the synthetic table above [changes are marked in red].

It shall be noted that we create this expanded dataset by reweighting each landmark subset then combining them into the stacked set.

Table S2: example data for Fine-Gray landmark supermodel

ID	Tstart [a]	Tstop [b]	status [c]	MS_is_ICU_unit	LAB_CRP_last	weight.cens	count	failcode
1	0	4.42	1	0	28.6	1	1	1
1	1	4.42	1	0	50.7	1	1	1
1	2	4.42	1	0	46.2	1	1	1
1	3	4.42	1	1	46.7	1	1	1
1	4	4.42	1	0	21.2	1	1	1
2	0	7.00	0	1	86.1	1	1	1
2	1	8.00	0	1	99.7	1	1	1
2	2	9.00	0	1	87.5	1	1	1
2	3	9.34	1	1	51.2	1	1	1
2	4	9.34	1	1	40.7	1	1	1
2	5	9.34	1	1	27.5	1	1	1
2	6	9.34	1	1	19.6	1	1	1
2	7	9.34	1	1	29.3	1	1	1
2	8	9.34	1	1	17.8	1	1	1
2	9	9.34	1	1	9.6	1	1	1
3	0	1.29	2	1	90.1	1	1	1
3	1.29	4.42	2	1	90.1	1	2	1
3	1	1.29	2	1	131.4	1	1	1
3	1.29	4.42	2	1	131.4	1	2	1
4	0	4.56	3	0	41.1	1	1	1
4	1	4.56	3	0	157.3	1	1	1
4	2	4.56	3	0	167.9	1	1	1
4	3	4.56	3	0	134.5	1	1	1
4	4.56	9.34	3	0	134.5	1	2	1
4	4	4.56	3	0	41.6	1	1	1
4	4.56	9.34	3	0	41.6	1	2	1

[a]Tstart = LM
[b]Tstop = eventtime
[c]status = type

In the above table, events of CLABSI was observed at 4.42 for landmark time 0 to 4 and at 9.34 for landmark time 3 to 9. In the expanded table, the first two individuals didn't change. However, the individual 3, who had a competing event Death at 1.29, is spread over several rows (actually, they are the time when CLABSI happens: 4.42). Same happens on individual 4. As the time when competing event Discharge happened at a later time (4.56) than 4.42, thus it is only spread over the rows when a later CLABSI happened (9.34). Due to administrative censoring that we applied here, censoring time can only be 7+LM, thus the weight did not change and was always 1.

Supplementary file 3: individual trajectories of time-varying continuous predictors

Figure S1 presents spaghetti plots for four time-varying continuous variables, visualized across time for a random sample of 50 patients. By following the longitudinal trajectories of these variables, we highlight their dynamic nature and inter-individual variability, which motivates the use of dynamic prediction models. We filtered only 50 patients to reduce overplotting and avoid clutter.

Figure S1: individual trajectories of time-varying continuous variables in a sample of 50 patients

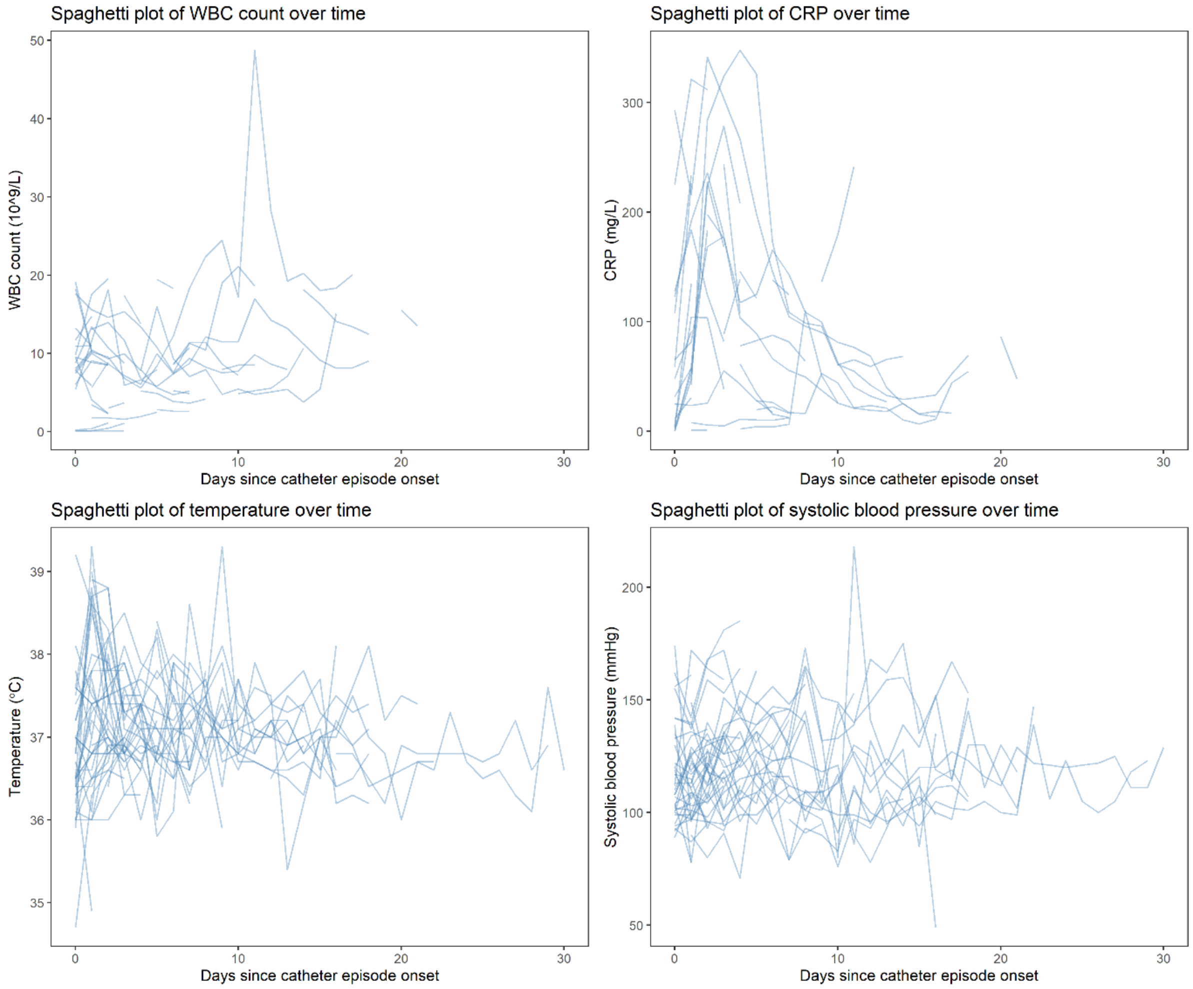

Supplementary file 4: methodology

- **Static models**

Logistic regression is used to model the event, which is an indicator of whether CLABSI occurred within the 7 days after catheter onset but ignoring when the event happened exactly. We used the data at catheter onset and related the probability of CLABSI occurring in an interval to a logistic function of the risk factors [5].

Let N be the number of individuals in the dataset, each with a set of k risk factors, $Z' = (z_1, z_2, \dots, z_k)$, measured at the beginning (catheter onset) of a follow-up period of length T (7 days). Defining a dichotomous response variable Y by

$$Y = \begin{cases} 1, & if\ the\ event\ of\ interest\ happens\ during\ follow\ up; \\ 0, & otherwise; \end{cases}$$

then the logistic function of risk factors Z with coefficients β_0 and β is given as follows:

$$P(Y = 1|Z) = \{1 + \exp[-\beta_0 - \beta' Z]\}^{-1} \quad (1)$$

The parameter β_0 is the intercept for the logistic model and the Z represent the covariates that are available at catheter onset.

When there are competing events, these can be taken into account in the outcome indicator. Assuming J+1 possible events, multinomial logistic regression is a generalized linear model used to estimate the probabilities for each event j = 0, 1, ... , J (assuming the reference level is event j=0). It models the probability of the 1st category (always assuming the event of interest is 1st category) of a dependent variable Y, using a set of explanatory variables Z [6]:

$$P(Y = 1|Z) = \frac{\exp(\beta_{01} + \beta_1' Z)}{1 + \sum_{j=1}^{J} \exp(\beta_{0j} + \beta_j' Z)} \ with\ j = 1, \dots, J \quad (2)$$

where β_j is the row vector of regression coefficients of Z for the jth category of Y. The category 0 denotes the main category, which, in this case study, refers to those patients who have not experienced any type of event excluding CLABSI, death or discharge.

When the outcome is of a time-to-event nature, survival models are commonly advised. The basic approach is the Cox proportional hazards model. We denote by T the time between the data of time zero (catheter onset) and the date of an event, the corresponding survival function is given by

$$S(T|Z, \alpha) = \exp\left(-\int_0^T \lambda_0(t) \exp(\beta' Z)\, dt\right) \quad (3)$$

where $\lambda_0(t)$ denotes baseline hazard, which refers to the probability that a person with all zero values for covariates will experience the event in the next instant if the person survives to *t*. Note that the coefficients vector β for the risk factors Z are not necessarily the same as the coefficients β' in (1).

Under competing risks setting, we denote by D ∈ {1, …, J} the different events. We assume that $\{D = 1\}$ is the event of interest. The absolute risk formula is given by [7]:

$$\mathrm{F}_1(\mathrm{T}|\mathrm{Z}) = \int_0^T S(t|Z) \lambda_1^{cs}(t) dt \quad (4)$$

where $\mathrm{F}_1(\mathrm{T}|\mathrm{Z})$ and $\lambda_1^{cs}(t)$ denotes the cumulative incidence function (CIF) and the cause-specific hazard rate respectively for the target event of interest. The absolute risk is determined as the accumulation over the time interval [0, T] of the product between the event-free survival and the

hazard of experiencing the event of interest, conditional to the covariates. The event-free survival can be estimated from the cause-specific hazards using:

$$S(\mathrm{T}|Z) = \exp\left(-\int_0^T \sum_{j=1}^{J} \lambda_j^{cs}(t|Z)\, dt\right) \tag{5}$$

where the hazard $\lambda_j^{cs}(t|Z)$ is termed proportional as it is the product of baseline hazard of its corresponding type of event $\lambda_{j0}^{cs}(t)$ and the corresponding function of the risk factors for the disease $\exp(\beta_j' Z)$.

Given the formula (4) and (5), it can be seen that the cause-specific CIF depends on the cause-specific hazard function for all the event types. In cause-specific hazards model, CIFs are estimated using Kaplan-Meier methods. While in Fine-Gray models, it models the effect of covariates on the CIF directly.

Fine-Gray model considers the subdistribution hazard function as [8]:

$$\lambda_{\mathrm{j}}^{\mathrm{sh}}(\mathrm{t}|\mathrm{Z}) = \lim_{\Delta\mathrm{t}\to 0} \frac{\mathrm{P}(\mathrm{T}<\mathrm{t}+\Delta\mathrm{t}, \mathrm{J}=\mathrm{j}|\mathrm{Z}\{\mathrm{T}\geq\mathrm{t} \text{ or } (\mathrm{T}<\mathrm{t} \text{ and } \mathrm{J}\neq\mathrm{j})\})}{\Delta\mathrm{t}} \tag{6}$$

The absolute risk of the event of interest can be obtained by:

$$\mathrm{F}_1(\mathrm{T}|\mathrm{Z}) = 1 - \exp\left(\int_0^T \lambda_1^{\mathrm{sh}}(\mathrm{t}|\mathrm{Z})dt\right) \tag{7}$$

All above mentioned survival models reply on the validity of the proportional hazards assumption. A potential solution to the robustness problem is “stopped Cox regression”, by using only the data that is directly associated to the survival probability *S(T|Z)* of interest [9]. One approach to address this is obtaining the survival probability provided that all observations are administratively censored at that target prediction horizon. This approach can be applied in the setting of competing risks, and also in the dynamic landmark models.

- **Dynamic models**

The landmark approach in survival analysis involves selecting specific time points, known as "landmarks," at which the risk estimates for an event of interest are updated, using the information on the individuals who survive up to that given landmark time point.

Let *w* be the prediction window of interest. We aim to create a model to estimate risk at landmark time *s*, knowing an individual’s covariates at *s*, namely *Z(s)*, conditioning on being alive at *s*. To create the landmark model, risk prediction times of interest are first partitioned into different landmarks $\{s_0, \dots, s_L\}$. The sliding landmark datasets are created for each landmark *s*, using only the data of individuals still at risk at *s*, and applying artificial censoring at *s*+*w* to these individuals.

Separate Cox model can be fitted at each landmark *s* for which a prediction is required. However, this is impractical and difficult to communicate with clinical users [4]. Some form of smoothing and simplification is needed. This can be achieved by computing all the separate prediction models and smoothing them by constructing a stacked super dataset. In this case, we assume the baseline hazard depends on *s* and this can be modelled by $\lambda_0(t|Z(s), s) = \lambda_0(t)\exp(\gamma(s))$, $where\ \gamma(s) = \gamma_1\left(\frac{s}{30}\right) + \gamma_2\left(\frac{s}{30}\right)^2$. Note that $\frac{s}{30}$ is applied here to constrain the effect sizes of γ_1 and γ_2. The landmark Cox supermodel can be fitted by applying a Cox proportional hazards model to the stacked dataset of the different landmarks and the predicted survival function is:

$$\begin{aligned} S(\mathrm{s} + w|Z(s), s) &= \exp\left(-\left[\widehat{\Lambda}(s + w|Z(s), s) - \widehat{\Lambda}(s|Z(s), s)\right]\right) \\ &= \exp\left(-\, exp\left(\gamma(s) + \beta(s)Z(s)\right)\left[\widehat{\Lambda_0}(s + w|Z(s), s) - \widehat{\Lambda_0}(s|Z(s), s)\right]\right) \end{aligned} \tag{8}$$

where $\widehat{\Lambda_0}$ is the cumulative hazard and its numerical integration can be calculated using the *evalstep* function in *dynpred* package [10]. The predicted risk, that is, cumulative incidence, can be obtained using

$$F(s+w|Z(s),s) = 1 - S(s+w|Z(s),s) \quad (9)$$

Similarly, we can fit landmark cause-specific supermodel and consider the baseline hazards $\lambda_{j0}(t)$ from each of the cause-specific Cox models for event J (J=1,…, j) as $\lambda_{j0}^{cs}(t|Z(s),s) = \lambda_{j0}^{cs}(t)\exp\left(\gamma_j(s)\right)$ and $\gamma_j(s) = \gamma_{j1}\left(\frac{s}{30}\right) + \gamma_{j2}\left(\frac{s}{30}\right)^2$. Thus, the cause-specific hazards of supermodel for event J from a landmark time $s \in [s_0, s_L]$ for time t ($s \le t \le s+w$) is:

$$\lambda_j^{cs}(t|Z(s),s) = \lambda_{j0}^{cs}(t)\exp\left(\gamma_j(s) + \beta_j(s)Z(s)\right) \quad (10)$$

Then w-year event free survival at any time point s in the window $[s_0, s_L]$ can be estimated with either the exponential approximation [11]:

$$S(s+w|Z(s),s) = \exp\left(-\int_s^{s+w}\sum_{j=1}^{J}\lambda_j^{cs}(t|Z(s),s)\,dt\right) = \exp\left(-\sum_{s\le t_i\le s+w}\sum_{j=1}^{J}\lambda_j^{cs}(t_i|Z(s),s)\right) \quad (11)$$

or the product integral estimator [11] when there are three competing events:

$$S(s+w|Z(s),s) = \prod\nolimits_{s\le t_i\le s+w}\left(1 - d\Lambda_1^{cs}(t_i|Z(s),s) - d\Lambda_2^{cs}(t_i|Z(s),s) - d\Lambda_3^{cs}(t_i|Z(s),s)\right) \quad (12)$$

where Λ_j^{cs} denotes the cumulative cause-specific hazard of event j. The cause-specific cumulative incidence for event J at any time point s in the window $[s_0, s_L]$ can be obtained with

$$F_j(s+w|Z(s),s) = \int_s^{s+w}\lambda_j^{cs}(t|Z(s),s)S(t|Z(s),s)\,dt = \sum_{s\le t_i\le s+w}\lambda_j^{cs}(t_i|Z(s),s)S(t_i|Z(s),s) \quad (13)$$

where t_i are event times in the training dataset used to fitting the landmark cause-specific supermodel.

The concern in developing a landmark supermodel based on the Fine-Gray approach was in constructing the landmark dataset at each landmark to properly account for competing events that happened before the landmark in the setting of subdistribution hazard [12]. Liu et al. extended the landmark method to the Fine-Gray model and proposed landmark proportional subdistribution hazards (PSH) model [13] and the target dynamic prediction probabilities $F_j(s+w|Z(s),s)$ can be estimated as

$$\begin{aligned} F_j(s+w|Z(s),s) &= 1 - \exp\left(-\left[\widehat{\Lambda_j^{sh}}(s+w|Z(s),s) - \widehat{\Lambda_j^{sh}}(s|Z(s),s)\right]\right) \\ &= 1 - \exp\left(-\exp\left(\gamma_j(s) + \beta_j Z(s)\right)\left[\widehat{\Lambda_{j0}^{sh}}(s+w|Z(s),s) - \widehat{\Lambda_{j0}^{sh}}(s|Z(s),s)\right]\right) \end{aligned} \quad (14)$$

where $\widehat{\Lambda_j^{sh}}$ is the Breslow estimator for the cumulative subdistribution hazard of event J.

To implement the model in a Fine-Gray approach and obtain the estimated cumulative subdistribution hazard, each subset needs to be transformed into the counting process style before stacking all landmark subsets into a super dataset. Also, time-varying inverse probability censored weighting (IPCW) needs to be calculated for the subjects who experienced competing risks [14], which can be achieved with the function *finegray* from *survival* package [15].

As we mentioned for the static model, logistic regression can be used to link predictors to the binary event outcome. We used our landmark datasets which treats it as repeated observations at landmark times and related the probability of CLABSI occurring in an interval to a logistic function of the risk factors [16].

$$\log\left(\frac{P(Z(s),s)}{1-P(Z(s),s)}\right) = \beta_0 + \gamma_{log}(s) + \beta Z(s) \qquad (15)$$

The parameter γ_{log} denotes the effect of landmark time and here we assume a linear and quadratic trend on the time effect as $\gamma_{log}(s) = \gamma_1\left(\frac{s}{30}\right) + \gamma_2\left(\frac{s}{30}\right)^2$. The parameters β_0 and $\beta(s)$ are the intercept and variable estimates for the logistic landmark supermodel respectively.

Similar to the landmark logistic regression , we applied the multinomial logistic regression on the landmark dataset. The event indicator is not restricted to binary (0/1) cases. Here we used categorical variable to indicate the event (1=CLABSI; 2=Death, 3=Discharge/catheter removal; 0=administrative censored within 7 days) in each interval. Thus the probability of CLABSI can be obtained via

$$\log\left(\frac{P(Y=1|Z(s),s)}{P(Y=0|Z(s),s)}\right) = \beta_{10} + \gamma_{multi}(s) + \beta_1 Z(s) \qquad (16)$$

The parameter γ_{multi} denotes the effect of landmark time on CLABSI compared to base category and here we assume a linear and quadratic trend on the time effect as $\gamma_{multi}(s) = \gamma_{11}\left(\frac{s}{30}\right) + \gamma_{12}\left(\frac{s}{30}\right)^2$. The parameters β_{10} and β_1 are like binary logistic regression expressing the relative log-odds of getting CLABSI compared to the base category.

Besides, we also implement regularized multi-task learning (MTL) for simultaneous learning of regression tasks on our stacked landmark datasets [17]. The algorithm follows the framework

$$\min_{W,C} \sum_{i=1}^{t} L(W_i, C_i|X_i, Y_i) + \lambda_1\Omega(W) + \lambda_2||W||_F^2 \qquad (17)$$

where X and Y are predictors matrices and responses of multiple tasks (landmarks) respectively. $L(W_i, C_i|X_i, Y_i)$ is the logistic loss function. W is the coefficient matrix. The algorithm incorporates not only the summation of logistic loss function across all landmarks, but also the cross-task regularization $\Omega(W)$ for knowledge transfer, and $||W||_F^2$ for improving the generalization. $\Omega(W)$ jointly modulates multi-tasks models W_i across all landmarks according to the specific prior structure. In this case we used network-based relatedness, that is, $||WG||_F^2$ for $\Omega(W)$, which equals to an accumulation of differences between related tasks. The different of connected task can be 0 if the penalty is heavy enough.

Table S3 and Table S4 provided summaries of prediction algorithms for the landmark approach models in both static and dynamic models.

Table S3: summary of the algorithms for static models

Model	Outcome	Prediction
binary logistic regression	$Y = \begin{cases} 1, & CLABSI \text{ occurred within 7 days;} \\ 0, & otherwise; \end{cases}$	$\{1 + \exp[-\beta_0 - \beta' Z]\}^{-1}$
multinomial logistic regression	$Y = \begin{cases} 1, & CLABSI \text{ occurred within 7 days;} \\ 2, & death \text{ occurred within 7 days;} \\ 3, & discharge/catheter\ removal \text{ within 7 days;} \\ 0, & otherwise; \end{cases}$	$\frac{\exp(\beta_{01}+\beta_1' Z)}{1+\sum_1^3 \exp\left(\beta_{0j}+\beta_j' Z\right)}$ $with\ j = 1, \dots, 3$
Cox proportional hazards model	[T, Y] where T is the failure time (when event occurs) and Y is the event indicator $Y = \begin{cases} 1, & CLABSI\ ; \\ 0, & otherwise; \end{cases}$	$1 - \exp\left(-\int_0^7 \lambda_0(t) \exp(\beta' Z)\, dt\right)$
cause-specific model	[T, Y] where T is the failure time (when event occurs) and Y is the event indicator $Y = \begin{cases} 1, & CLABSI; \\ 2, & death; \\ 3, & discharge/catheter\ removal; \end{cases}$	$\int_0^T \exp\left(-\int_0^7 \sum_{j=1}^{J} \lambda_j^{cs}(t\|Z)\, dt\right) \lambda_1^{cs}(t) dt$
Fine-Gray model	[T, Y] where T is the failure time (when event occurs) and Y is the event indicator $Y = \begin{cases} 1, & CLABSI; \\ 2, & death; \\ 3, & discharge/catheter\ removal; \end{cases}$	$1 - exp\ (-\int_0^7 \lambda_1^{sh}(t\|Z) dt)$

Note: $Z' = (z_1, z_2, \dots, z_k)$ is the set of k risk factors, measured at the beginning (catheter onset) of follow-up period of length T. The parameter β_0 and β is the intercept and coefficient estimates for the logistic model. β_j is the row vector of regression coefficients of Z for the jth category of Y in multinomial logistic model. $\lambda_0(t)$ denotes the baseline hazard of Cox model, which refers to the probability that a person with all zero values for covariates will experience the event in the next instant if the person survives to t. Note that the coefficients vector β for the risk factors Z are not necessarily the same as the coefficients β' in binary logistic model. $\lambda_j^{cs}(t)$ and $\lambda_j^{sh}(t)$ denote the cause-specific and subdistribution hazard respectively for the corresponding event j.

Table S4: summary of the algorithms for dynamic models

Model	Outcome	Prediction
binary logistic landmark supermodel	$Y = \begin{cases} 1, & CLABSI \text{ occurred within } s+w \text{ days;} \\ 0, & otherwise; \end{cases}$	$\log\left(\frac{P(Z(s),s)}{1-P(Z(s),s)}\right) = \beta_0 + \gamma_{log}(s) + \beta Z(s)$
regularized multi-task learning (RMTL)	$Y = \begin{cases} 1, & CLABSI \text{ occurred within } s+w \text{ days;} \\ -1, & otherwise; \end{cases}$	$\log\left(\frac{P(Z(s))}{1-P(Z(s))}\right) = w_s Z^*(s)$ where w_s are the coefficients at each landmark s and $Z^*(s)$ represents the standardized covariates at s
multinomial logistic landmark	$Y = \begin{cases} 1, & CLABSI \text{ occurred within } s+w \text{ days;} \\ 2, & death \text{ occurred within } s+w \text{ days;} \\ 3, & discharge/catheter\ removal \text{ within } s+w \text{ days;} \\ 0, & otherwise; \end{cases}$	$\log\left(\frac{P(Y=1\|Z(s),s)}{P(Y=0\|Z(s),s)}\right) = \beta_{10} + \gamma_{multi}(s) + \beta_1 Z(s)$
Cox landmark supermodel	[T*, Y] where T is the administratively censored failure time T* = min(T, C) where C is the administrative censoring time $(s+w)$ and Y is the event indicator $Y = \begin{cases} 1, & CLABSI \text{ within } s+w \text{ days;} \\ 0, & otherwise; \end{cases}$	$1 - \exp\left(-\left[\hat{\Lambda}(s+w\|Z(s),s) - \hat{\Lambda}(s\|Z(s),s)\right]\right)$
cause-specific landmark supermodel	[T*, Y] where T is administratively censored failure time T* = min(T, C) where C is the administrative censoring time $(s+w)$ and Y is the event indicator $Y = \begin{cases} 1, & CLABSI \text{ within } s+w \text{ days;} \\ 2, & death \text{ within } s+w \text{ days;} \\ 3, & discharge/catheter\ removal \text{ within } s+w \text{ days;} \\ 0, & otherwise \end{cases}$	$\sum_{s \le t_i \le s+w} \lambda_1^{cs}(t_i\|Z(s),s) S(t_i\|Z(s),s)$
Fine-Gray landmark supermodel	[T*, Y] where T is administratively censored failure time T* = min(T, C) where C is the administrative censoring time $(s+w)$ Y is the event indicator $Y = \begin{cases} 1, & CLABSI \text{ within } s+w \text{ days;} \\ 2, & death \text{ within } s+w \text{ days;} \\ 3, & discharge/catheter\ removal \text{ within } s+w \text{ days;} \\ 0, & otherwise \end{cases}$	$1 - \exp\left(-\left[\widehat{\Lambda_1^{sh}}(s+w\|Z(s),s) - \widehat{\Lambda_1^{sh}}(s\|Z(s),s)\right]\right)$

Note: In logistic landmark supermodels, the parameter γ denotes the effect of landmark time and here we assume a linear and quadratic trend on the time effect as $\gamma_{log}(s) = \gamma_1\left(\frac{s}{30}\right) + \gamma_2\left(\frac{s}{30}\right)^2$. The parameters β_0 and β are the intercept and variable estimates for the logistic landmark supermodel respectively. Similarly, $\gamma_{multi}(s)$ denotes the effect of landmark time on CLABSI compared to base category (censored cases) and here we assume a linear and quadratic trend on the time effect as $\gamma_{multi}(s) = \gamma_{11}\left(\frac{s}{30}\right) + \gamma_{12}\left(\frac{s}{30}\right)^2$. The parameters β_{10} and β_1 are like binary logistic regression expressing the relative log-odds of getting CLABSI compared to the base category. In survival framework, we assume the baseline hazard depends on *s* and can be modelled by $\lambda_0(t|Z(s),s) = \lambda_0(t)\exp(\gamma(s)), where\ \gamma(s) = \gamma_1\left(\frac{s}{30}\right) + \gamma_2\left(\frac{s}{30}\right)^2$. Note that $\frac{s}{30}$ is applied here to constrain the effect sizes of γ_1 and γ_2. $\hat{\Lambda}$ is the cumulative hazard when there are no competing risks, and $\widehat{\Lambda_j^{sh}}$ is the Breslow estimator for the cumulative subdistribution hazard of event J in Fine-Gray framework. In cause-specific landmark supermodel, the cause-specific hazards for event J from a landmark time $s \in [s_0, s_L]$ for time *t* ($s \le t \le s+w$) is $\lambda_j^{cs}(t|Z(s),s)$ and can be obtained via $\lambda_{j0}^{cs}(t)\exp(\gamma_j(s) + \beta_j(s)Z(s))$ where $\lambda_{j0}^{cs}(t)$ denotes the baseline hazards from each of the cause-specific models for event J (J=1,2,3) and $\gamma_j(s)$ also assumes a linear and quadratic effect on $\left(\frac{s}{30}\right)$.

Supplementary file 5: missing data

Table S5: missing data

Column	Description	Variable	Missing at LM0	Missing at all LMs
Central venous catheter (CVC)	Was there a catheter of type CVC connected since previous LM?	time-varying	0.0%	0.0%
Port a cath	Was there a catheter of type Port_a_cath connected since previous LM?	time-varying	0.0%	0.0%
Tunneled central venous catheter	Was there a catheter of type tunneled CVC connected since previous LM	time-varying	0.0%	0.0%
Peripherally inserted central catheter (PICC)	Was there a catheter of type PICC connected since previous LM?	time-varying	0.0%	0.0%
Subclavian	Was a catheter connected in the Subclavian location since previous LM?	time-varying	0.0%	0.0%
Jugular	Was a catheter connected in the Jugular location since previous LM?	time-varying	0.0%	0.0%
Total parental nutrition (TPN)	Has TPN been ordered for the patient in the previous 7 days from LM time	time-varying	0.0%	0.0%
Antineoplastic agents	Have any drugs in ATC group (level 2) L01 (ANTINEOPLASTIC AGENTS) been ordered for the patient in the previous 7 days from LM time	time-varying	0.0%	0.0%
Antibacterials for systematic use	Have any drugs in ATC group (level 2) J01 (ANTIBACTERIALS FOR SYSTEMIC USE) been ordered for the patient in the previous 7 days from LM time	time-varying	0.0%	0.0%
CLABSI history	Did the patient experience a CLABSI event in the past 3 months since LM time?	time-varying	0.0%	0.0%
Tumor	Has a tumour pathology been registered before current LM time?	time-varying	0.0%	0.0%
Lymphoma	Has lymphoma been registered as a comorbidity before current LM time?	time-varying	0.0%	0.0%
Transplant	Has a transplant pathology been registered before current LM time?	time-varying	0.0%	0.0%
ICU unit	Is the patient now (at the exact second of the current LM) in ICU?	time-varying	0.0%	0.0%
Mechanical ventilation (MV)	Is the patient on MV since previous landmark? A patient is considered on MV if at least one value of PEEP or FiO2 are recorded between 2 landmarks. Only valid for ICU patients.	time-varying	0.0%	0.0%
Temperature	Maximum value of temperature since previous landmark. At LM 0 (catheter onset) the last value from the previous 24 hours is used. Only temperatures in the range (30 °C, 45 °C) are kept, the others are filtered. Maximum value is used to correct for very low temperatures measured by devices in ICU, when the temperature falls closer to the room temperature.	time-varying	26.7%	10.9%
Systolic blood pressure	Last value of systolic blood pressure since previous landmark. At LM 0 (catheter onset) the last value from the previous 24 hours is used.	time-varying	28.0%	15.3%
White blood cells (WBC) count	WBC count, last value since previous LM. Unit: 10**9/L	time-varying	41.7%	39.4%
C-reactive protein (CRP)	CRP, last value since previous LM. Unit: mg/L	time-varying	46.7%	40.5%
Positive culture, of any other type than blood	Has there been a positive culture, of any other type than blood, in the last 17 days (time window used for secondary BSIs).	time-varying	0.0%	0.0%

Patients admitted from home	Was the patient admitted from home?	catheter onset	1.6%	not applicable

Supplementary file 6: sample size calculation

We used the pmsampsize package in R to estimate the minimum sample size required for developing a static logistic regression model and a static Cox proportional hazards [18]. According to a systematic review of CLABSI prediction models [19], the mean of optimism-corrected AUROC is 0.75 in the studies with similar EHR setting. Assuming we can obtain a c-statistic of 0.75 with 21 parameters and a prevalence of 3.14%, a minimum of 7,027 and 6,974 unique catheter episodes are required to develop logistic and Cox models, respectively. When the prevalence is 1.31%, at least 16,621 (logistic) and 15,912 (Cox) catheter episodes are required. Hence, our sample size of 30,862 is sufficiently large for static models if applying a 2:1 train test split. Given the utilization of the landmark approach for dynamic models, where information from adjacent landmarks is leveraged, we conclude that the same sample size rationale is applicable for dynamic models as well.

Supplementary file 7: codes

The codes are available via this link: https://github.com/chendcw/CLABSI_compare_models.

Supplementary file 8: summary of characteristics at catheter onset

Table S6: summary of patient characteristics at catheter onset by outcome and overall

Variable Category	Variables	Statistics	CLABSI	Death	Discharge	Total
**catheter types**	Central venous catheter	n (%)	630 (64.9)	594 (40.5)	12374 (43.5)	13598 (44.1)
	Port-a-cath	n (%)	202 (20.8)	722 (49.2)	13083 (46.0)	14007 (45.4)
	Tunneled central venous catheter	n (%)	100 (10.3)	40 (2.7)	1233 (4.3)	1373 (4.4)
	Peripherally inserted central catheter	n (%)	29 (3.0)	86 (5.9)	1168 (4.1)	1283 (4.2)
**catheter location**	Subclavian	n (%)	389 (40.1)	874 (59.6)	15495 (54.5)	16758 (54.3)
	Jugular	n (%)	522 (53.8)	440 (30.0)	11107 (39.1)	12069 (39.1)
**medication**	Total parenteral nutrition	n (%)	284 (29.3)	174 (11.9)	1910 (6.7)	2368 (7.7)
	Antibacterials for systematic use	n (%)	691 (71.2)	903 (61.6)	16208 (57.0)	17802 (57.7)
	Antineoplastic agents	n (%)	73 (7.5)	56 (3.8)	6013 (21.2)	6142 (19.9)
**CLABSI history**	History of CLABSI	n (%)	30 (3.1)	32 (2.2)	496 (1.7)	558 (1.8)
**comorbidity**	Tumor	n (%)	372 (38.4)	832 (56.8)	15580 (54.8)	16784 (54.4)
	Lymphoma	n (%)	42 (4.3)	79 (5.4)	1261 (4.4)	1382 (4.5)
	Transplant	n (%)	66 (6.8)	75 (5.1)	1366 (4.8)	1507 (4.9)
**physical ward**	ICU unit	n (%)	287 (29.6)	493 (33.6)	4275 (15.0)	5055 (16.4)
**care modules**	Mechanical ventilation	n (%)	98 (10.1)	213 (14.5)	1199 (4.2)	1510 (4.9)
	Temperature	median (IQR)	36.9 (36.5 to 37.4)	36.8 (36.4 to 37.4)	36.7 (36.4 to 37.2)	36.7 (36.4 to 37.2)
		range (min, max)	(31.6, 40.5)	(31.0, 41.0)	(30.5, 41.0)	(30.5, 41.0)
	Systolic blood pressure	median (IQR)	121.0 (105.2 to 138.8)	120.0 (104.0 to 138.0)	124.0 (111.0 to 140.0)	124.0 (110.0 to 140.0)
		range (min, max)	(51.0, 240.0)	(36.0, 231.0)	(40.0, 293.0)	(36.0, 293.0)
**laboratory test**	WBC count	median (IQR)	8.6 (5.6 to 12.2)	9.5 (6.2 to 14.3)	8.2 (5.7 to 11.3)	8.3 (5.7 to 11.5)
		range (min, max)	(0.1, 212.2)	(0.1, 203.0)	(0.1, 388.4)	(0.1, 388.4)
	CRP	median (IQR)	29.2 (4.7 to 72.6)	64.6 (23.0 to 140.4)	16.3 (2.7 to 57.7)	18.6 (3.0 to 63.4)
		range (min, max)	(0.3, 560.6)	(0.3, 587.2)	(0.3, 585.9)	(0.3, 587.2)
	Positive culture, of any other type than blood	n (%)	123 (12.7)	214 (14.6)	2757 (9.7)	3094 (10.0)
**Admission**	Whether patients admitted from: home (vs other places)	n (%)	725 (75.1)	1107 (76.3)	24805 (88.8)	26637 (87.7)

median (IQR) for continuous variables, before missing data imputation; n (%) for categorical variables. Patients can have two or more different catheter types simultaneously, therefore catheter type has been coded as a binary rather than a categorical variable with multiple categories. IQR = interquartile range

Supplementary file 9: performance of static models

Figure S2: AUROC plots of all static models for 100 train-test splits

(a) Cox-ac

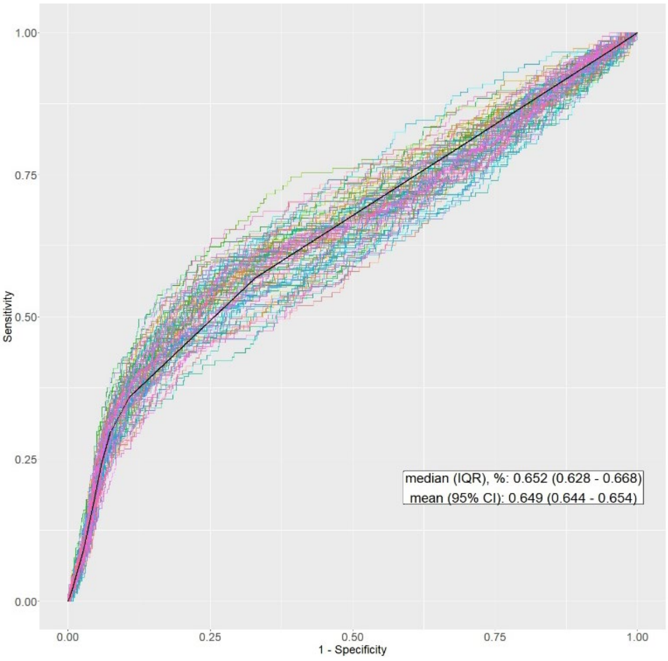

(b) Cox

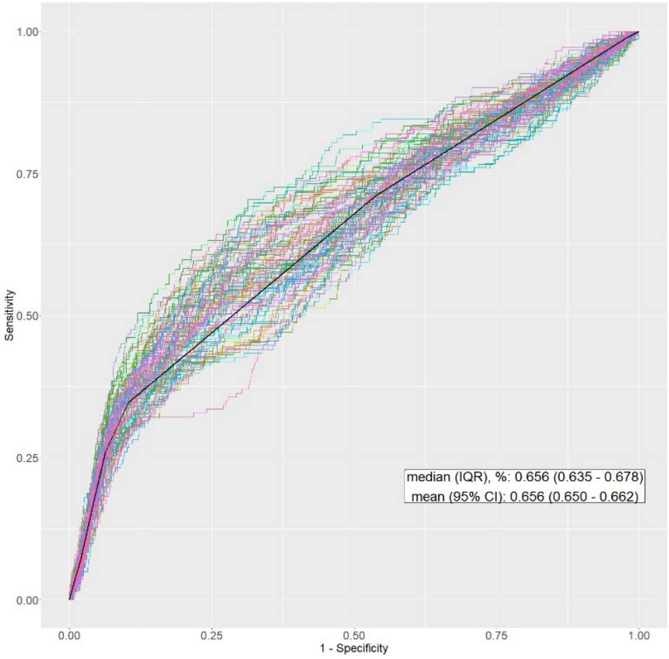

(c) CS-ac

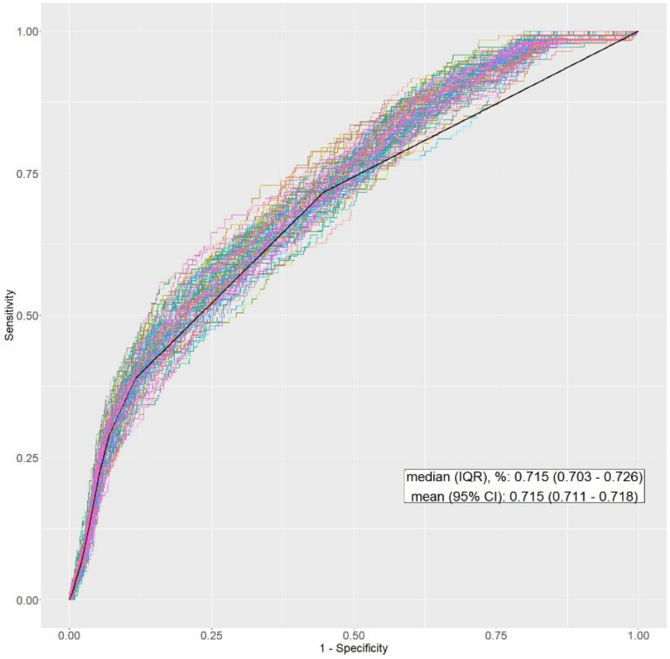

(d) CS

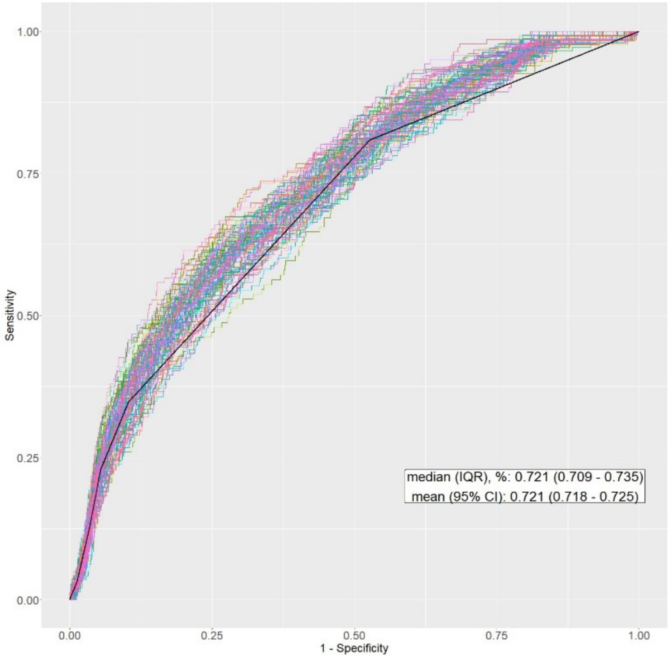

(e) FG-ac

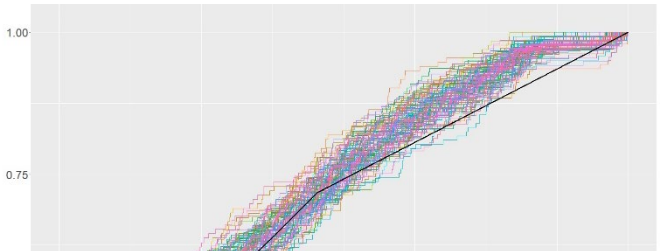

(f) FG

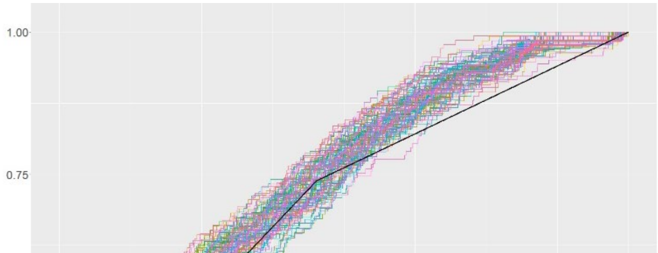

(g) LG

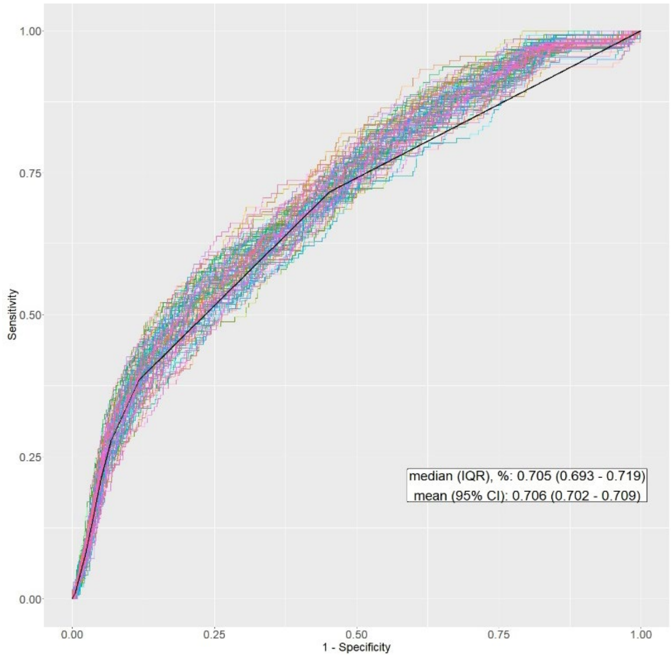

(h) MLR

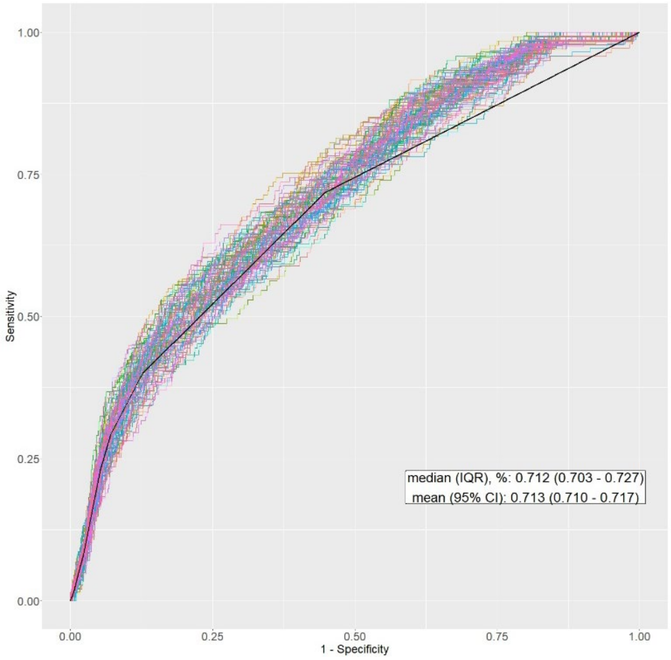

Figure S3: Calibration plots of all static models for 100 train-test splits

(a) Cox-ac

(b) Cox

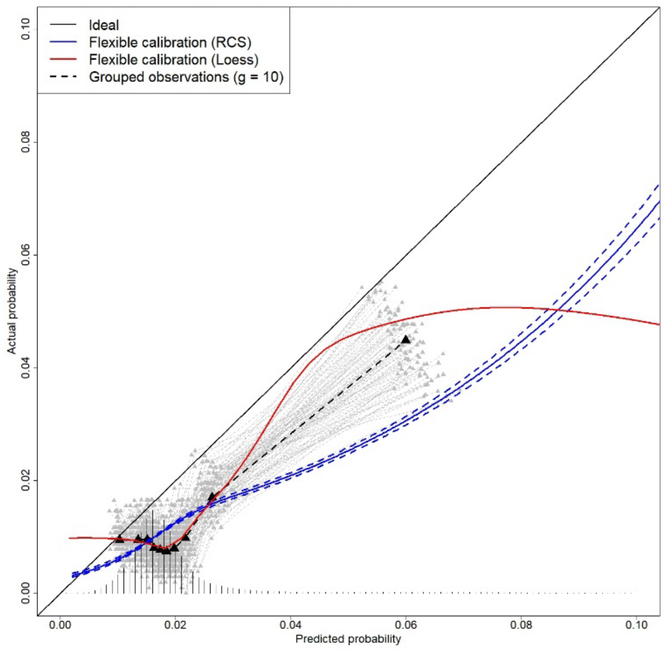

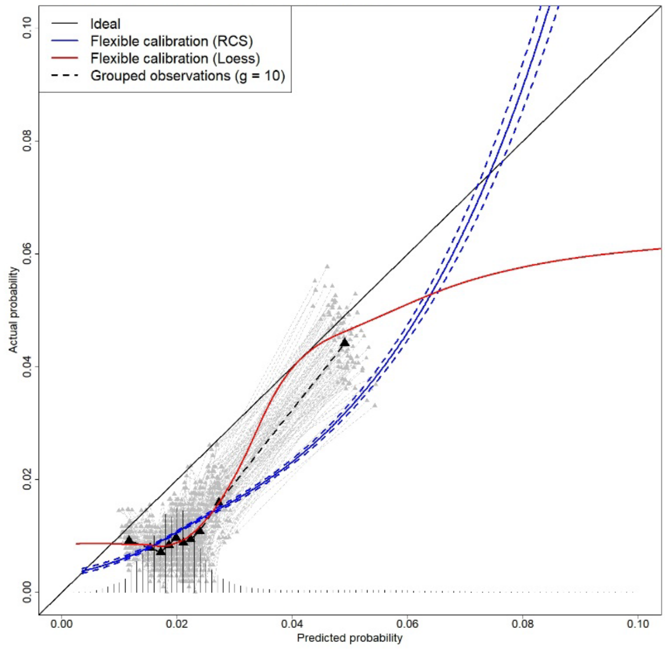

(c) CS-ac

(d) CS

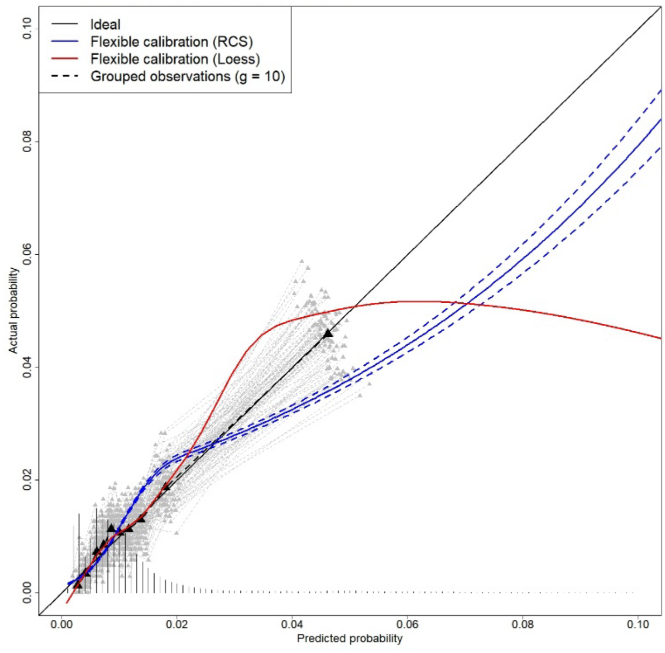

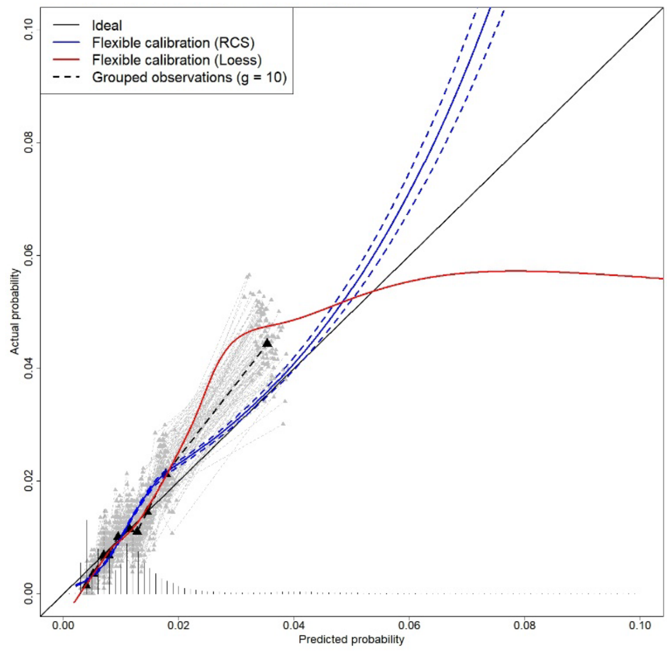

(e) FG-ac

(f) FG

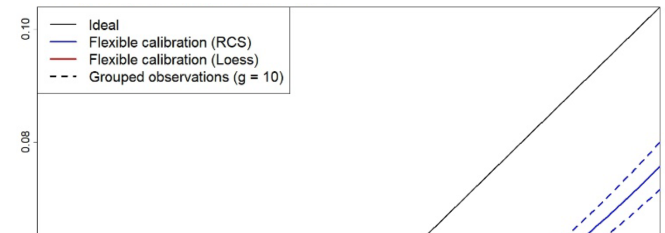

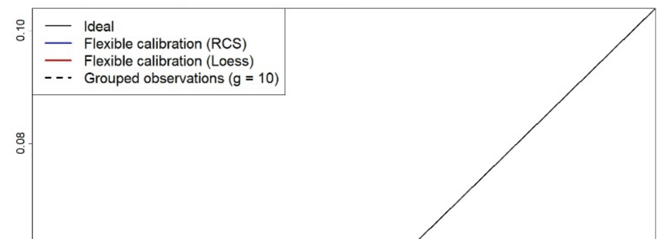

(g) LG

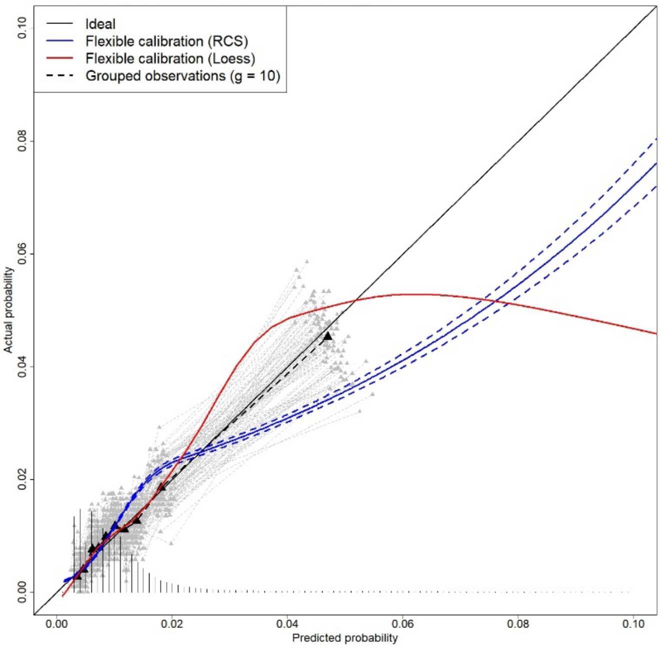
Ideal
Flexible calibration (RCS)
Flexible calibration (Loess)
Grouped observations (g = 10)
Actual probability
Predicted probability
0.00
0.02
0.04
0.06
0.08
0.10

(h) MLR

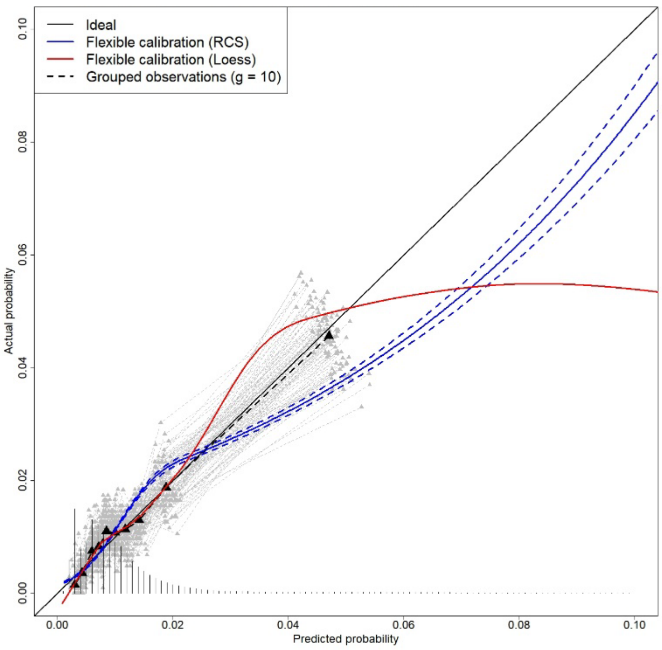
Ideal
Flexible calibration (RCS)
Flexible calibration (Loess)
Grouped observations (g = 10)
Actual probability
Predicted probability
0.00
0.02
0.04
0.06
0.08
0.10

Supplementary file 10: coefficient estimates

Figure S4: Coefficient estimates of the static models

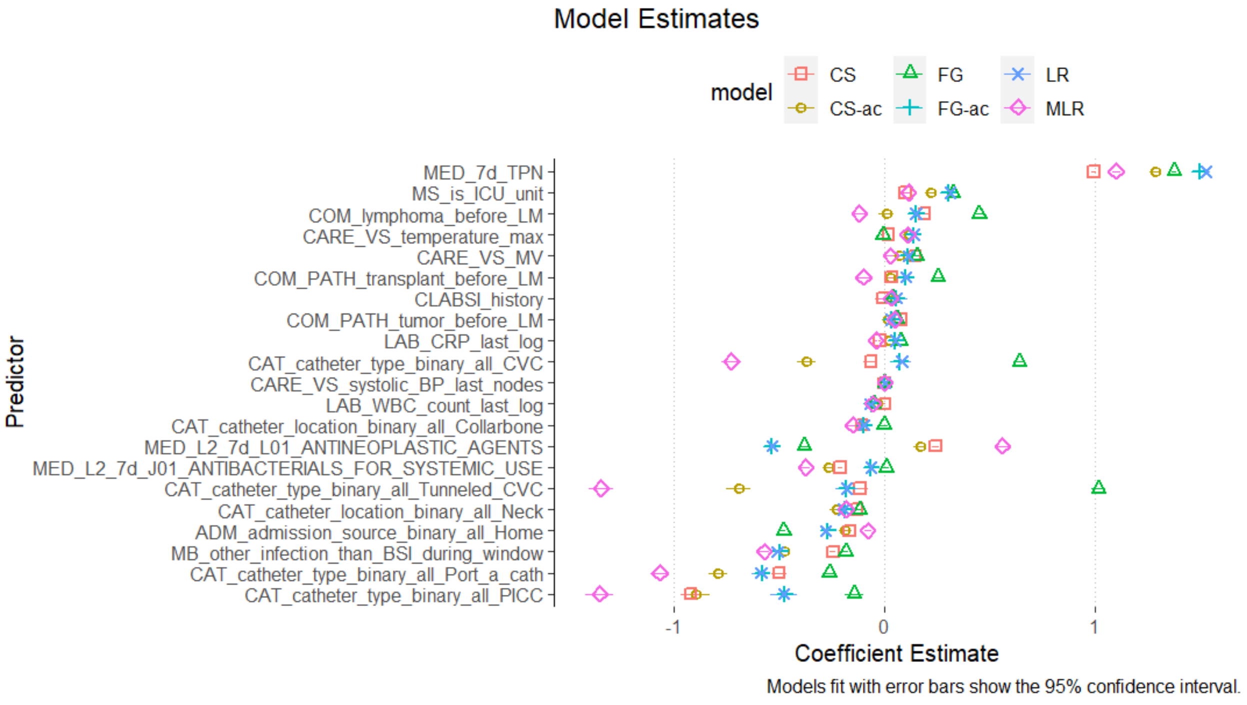

Note: the coefficient estimates of cox models (administrative censoring at day 7 and without administrative censoring) are not shown as they are the same as cause-specific models.

Table S7: coefficient estimates of static models

	CS-ac	CS	FG-ac	FG	LR	MLR
Patients admitted from home	-0.187 (-0.204, -0.17)	-0.166 (-0.178, -0.155)	-0.27 (-0.287, -0.253)	-0.479 (-0.491, -0.468)	-0.279 (-0.296, -0.261)	-0.08 (-0.097, -0.062)
Mechanical ventilation	0.071 (0.044, 0.098)	0.142 (0.124, 0.16)	0.108 (0.081, 0.135)	0.154 (0.137, 0.172)	0.112 (0.084, 0.139)	0.029 (0.002, 0.056)
Systolic blood pressure	0.001 (0, 0.001)	0 (0, 0)	0.001 (0, 0.001)	-0.001 (-0.001, -0.001)	0.001 (0, 0.001)	0.001 (0, 0.001)
Temperature	0.114 (0.104, 0.124)	0.018 (0.012, 0.025)	0.135 (0.125, 0.145)	-0.008 (-0.015, -0.001)	0.137 (0.127, 0.148)	0.111 (0.1, 0.121)
Catheter location: Subclavian	-0.107 (-0.138, -0.077)	-0.109 (-0.13, -0.087)	-0.099 (-0.128, -0.07)	-0.003 (-0.024, 0.018)	-0.097 (-0.127, -0.067)	-0.151 (-0.183, -0.12)
Catheter location: Jugular	-0.23 (-0.259, -0.201)	-0.131 (-0.153, -0.109)	-0.191 (-0.218, -0.164)	-0.118 (-0.138, -0.098)	-0.195 (-0.223, -0.168)	-0.182 (-0.211, -0.154)
Catheter type: CVC	-0.371 (-0.417, -0.325)	-0.065 (-0.099, -0.032)	0.073 (0.03, 0.116)	0.64 (0.612, 0.669)	0.081 (0.037, 0.125)	-0.728 (-0.775, -0.681)
Catheter type: PICC	-0.896 (-0.96, -0.831)	-0.922 (-0.966, -0.878)	-0.476 (-0.537, -0.415)	-0.145 (-0.187, -0.102)	-0.479 (-0.541, -0.416)	-1.353 (-1.42, -1.286)
Catheter type: Port_a_cath	-0.791 (-0.837, -0.745)	-0.502 (-0.539, -0.466)	-0.582 (-0.625, -0.54)	-0.263 (-0.294, -0.231)	-0.586 (-0.629, -0.544)	-1.068 (-1.114, -1.022)
Catheter type: Tunneled CVC	-0.694 (-0.752, -0.637)	-0.116 (-0.153, -0.079)	-0.183 (-0.235, -0.131)	1.016 (0.985, 1.047)	-0.18 (-0.233, -0.127)	-1.347 (-1.404, -1.29)
CLABSI history	0.051 (0, 0.103)	-0.01 (-0.045, 0.025)	0.055 (0.004, 0.107)	0.037 (0.004, 0.07)	0.056 (0.004, 0.109)	0.031 (-0.021, 0.083)
Lymphoma	0.009 (-0.026, 0.044)	0.189 (0.168, 0.21)	0.149 (0.114, 0.183)	0.448 (0.427, 0.47)	0.148 (0.113, 0.183)	-0.121 (-0.157, -0.085)
Transplant	0.029 (-0.002, 0.06)	0.033 (0.014, 0.051)	0.099 (0.067, 0.13)	0.254 (0.235, 0.273)	0.101 (0.069, 0.133)	-0.101 (-0.133, -0.069)
Tumor	0.017 (-0.004, 0.038)	0.078 (0.064, 0.091)	0.032 (0.01, 0.054)	0.06 (0.046, 0.074)	0.029 (0.007, 0.051)	0.047 (0.025, 0.069)
CRP (unit: mg/L)	0.021 (0.016, 0.027)	-0.023 (-0.027, -0.02)	0.051 (0.046, 0.057)	0.077 (0.073, 0.081)	0.052 (0.047, 0.058)	-0.041 (-0.047, -0.035)
WBC count (unit: 10^9/L)	-0.037 (-0.051, -0.022)	-0.002 (-0.01, 0.006)	-0.063 (-0.078, -0.048)	-0.053 (-0.062, -0.044)	-0.066 (-0.082, -0.051)	-0.055 (-0.071, -0.04)
Positive culture	-0.474 (-0.497, -0.452)	-0.245 (-0.258, -0.231)	-0.499 (-0.521, -0.476)	-0.186 (-0.199, -0.172)	-0.51 (-0.533, -0.487)	-0.572 (-0.595, -0.55)
TPN	1.29 (1.272, 1.308)	0.995 (0.984, 1.006)	1.495 (1.476, 1.513)	1.376 (1.365, 1.387)	1.527 (1.508, 1.546)	1.1 (1.082, 1.119)
Antibacterials for systematic use	-0.265 (-0.287, -0.244)	-0.211 (-0.223, -0.2)	-0.065 (-0.087, -0.042)	0.008 (-0.005, 0.021)	-0.064 (-0.087, -0.041)	-0.375 (-0.397, -0.352)
Antineoplastic agents	0.17 (0.14 , 0.199)	0.243 (0.226 , 0.261)	-0.537 (-0.57 , -0.505)	-0.383 (-0.403 , -0.363)	-0.534 (-0.567 , -0.502)	0.559 (0.528 , 0.591)
ICU_unit	0.221 (0.2 , 0.242)	0.098 (0.084 , 0.111)	0.301 (0.28 , 0.322)	0.326 (0.312 , 0.339)	0.31 (0.289 , 0.332)	0.113 (0.092 , 0.135)

Figure S5: Coefficient estimates of the dynamic models

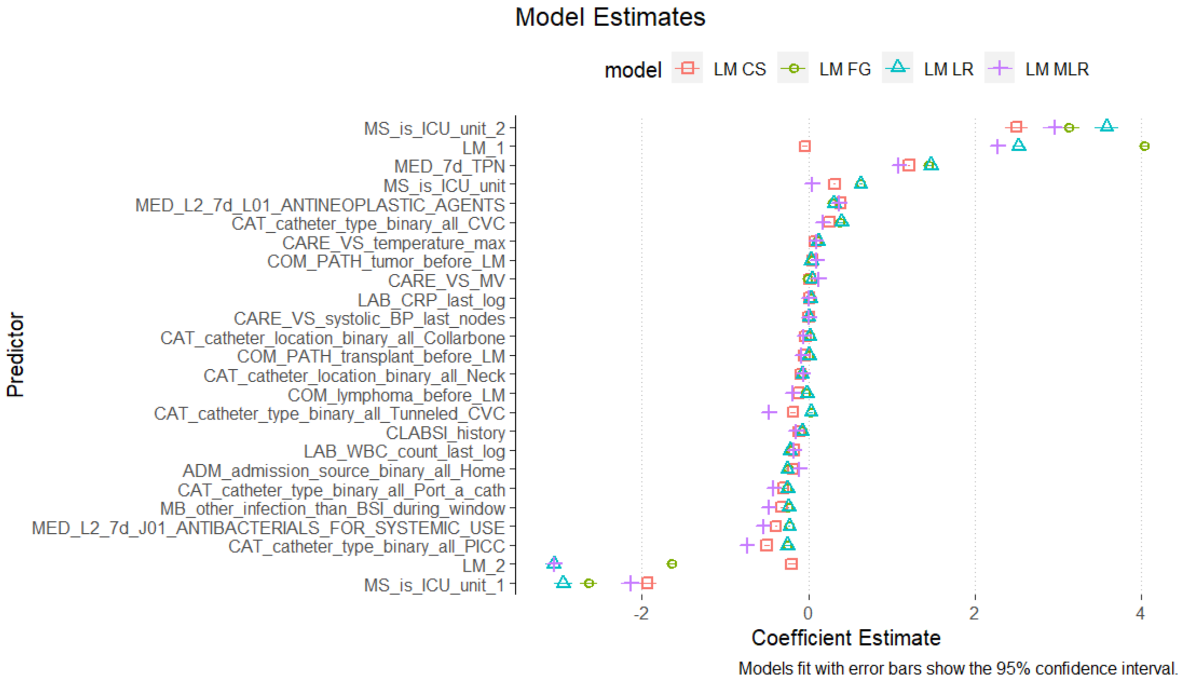

Note: the coefficient estimates of separate logistic landmark model and RMTL model are not shown as they have different coefficients across all landmarks; the coefficient estimates of cox landmark supermodels is not shown as they are the same as cause-specific landmark supermodel.

Table S8: coefficient estimates of dynamic models

	LM CS	LM FG	LM LR	LM MLR
Patients admitted from home	-0.183 (-0.196, -0.17)	-0.245 (-0.258, -0.232)	-0.256 (-0.269, -0.243)	-0.108 (-0.121, -0.094)
Mechanical ventilation	0.019 (0.007, 0.03)	-0.003 (-0.014, 0.008)	0.041 (0.03, 0.053)	0.119 (0.108, 0.131)
Systolic blood pressure	0.002 (0.002, 0.002)	0.003 (0.002, 0.003)	0.003 (0.003, 0.003)	0.002 (0.002, 0.003)
Temperature	0.085 (0.08, 0.089)	0.112 (0.107, 0.117)	0.118 (0.113, 0.123)	0.091 (0.086, 0.096)
Catheter location: Subclavian	-0.034 (-0.048, -0.02)	0.012 (-0.001, 0.026)	0.015 (0.001, 0.028)	-0.055 (-0.069, -0.04)
Catheter location: Jugular	-0.094 (-0.109, -0.078)	-0.074 (-0.089, -0.06)	-0.074 (-0.089, -0.059)	-0.064 (-0.08, -0.048)
Catheter type: CVC	0.252 (0.227, 0.277)	0.379 (0.355, 0.403)	0.398 (0.373, 0.422)	0.167 (0.142, 0.192)
Catheter type: PICC	-0.499 (-0.533, -0.464)	-0.259 (-0.293, -0.225)	-0.252 (-0.286, -0.218)	-0.741 (-0.775, -0.706)
Catheter type: Port_a_cath	-0.302 (-0.328, -0.276)	-0.257 (-0.281, -0.232)	-0.253 (-0.278, -0.227)	-0.418 (-0.443, -0.393)
Catheter type: Tunneled CVC	-0.186 (-0.214, -0.157)	0.028 (0.001, 0.055)	0.031 (0.003, 0.059)	-0.477 (-0.506, -0.449)
CLABSI history	-0.116 (-0.15, -0.081)	-0.079 (-0.113, -0.045)	-0.078 (-0.113, -0.043)	-0.15 (-0.186, -0.114)
Lymphoma	-0.12 (-0.143, -0.097)	-0.035 (-0.058, -0.011)	-0.018 (-0.042, 0.006)	-0.189 (-0.214, -0.165)
Transplant pathology	-0.051 (-0.071, -0.03)	-0.001 (-0.022, 0.019)	0.007 (-0.015, 0.028)	-0.084 (-0.105, -0.064)
Tumor pathology	0.047 (0.034, 0.06)	0.037 (0.024, 0.051)	0.035 (0.021, 0.048)	0.098 (0.085, 0.112)
CRP (unit: mg/L)	0.01 (0.007, 0.014)	0.024 (0.02, 0.027)	0.028 (0.024, 0.032)	0.006 (0.003, 0.01)
WBC count (unit: 10^9/L)	-0.171 (-0.177, -0.166)	-0.213 (-0.219, -0.208)	-0.22 (-0.226, -0.214)	-0.171 (-0.177, -0.166)
landmark/30	-0.043 (-0.077, -0.01)	4.04 (3.999, 4.082)	2.521 (2.449, 2.593)	2.272 (2.199, 2.344)
(landmark/30)^2	-0.201 (-0.246, -0.157)	-1.639 (-1.692, -1.585)	-3.06 (-3.154, -2.966)	-3.049 (-3.144, -2.954)
Positive culture	-0.322 (-0.332, -0.312)	-0.236 (-0.246, -0.227)	-0.24 (-0.25, -0.23)	-0.481 (-0.492, -0.471)
TPN	1.208 (1.197, 1.219)	1.44 (1.429, 1.451)	1.473 (1.461, 1.484)	1.085 (1.073, 1.096)
Antibacterials for systematic use	-0.391 (-0.404, -0.378)	-0.232 (-0.245, -0.219)	-0.229 (-0.242, -0.216)	-0.544 (-0.558, -0.53)
Antineoplastic agents	0.388 (0.373, 0.404)	0.283 (0.268, 0.299)	0.304 (0.288, 0.32)	0.364 (0.348, 0.38)
ICU_unit	0.314 (0.296, 0.332)	0.632 (0.614, 0.651)	0.625 (0.607, 0.644)	0.045 (0.027, 0.063)
ICU_unit * landmark/30	-1.93 (-2.037, -1.823)	-2.642 (-2.749, -2.534)	-2.947 (-3.061, -2.834)	-2.134 (-2.248, -2.02)
ICU_unit * (landmark/30)^2	2.5 (2.369, 2.63)	3.131 (3, 3.262)	3.582 (3.439, 3.725)	2.956 (2.811, 3.1)

Supplementary file 11: comparison of static and dynamic models with inclusion of age

Table S9: summary of the performance measures (mean with 95% CI) for static models with inclusion of age

Model	AUROC	Calibration Slope	O/E ratio	ECI	Scaled Brier
**Cox_age-ac**	0.655 (0.651, 0.660)	1.056 (1.026, 1.085)	0.598 (0.585, 0.611)	0.016 (0.014, 0.017)	0.000 (-0.001, 0.001)
**Cox_age**	0.654 (0.650, 0.659)	1.379 (1.346, 1.411)	0.575 (0.562, 0.587)	0.014 (0.013, 0.015)	0.000 (-0.001, 0.000)
**CS_age-ac**	0.712 (0.709, 0.716)	0.927 (0.906, 0.948)	1.015 (0.994, 1.037)	0.005 (0.004, 0.006)	0.008 (0.007, 0.008)
**CS_age**	0.720 (0.716, 0.723)	1.187 (1.164, 1.210)	1.039 (1.017, 1.060)	0.003 (0.003, 0.003)	0.009 (0.009, 0.010)
**FG_age-ac**	0.703 (0.699, 0.707)	0.896 (0.875, 0.917)	0.997 (0.976, 1.018)	0.005 (0.005, 0.006)	0.006 (0.006, 0.007)
**FG_age**	0.717 (0.714, 0.721)	0.863 (0.847, 0.878)	0.998 (0.977, 1.018)	0.006 (0.005, 0.006)	0.004 (0.004, 0.005)
**LR_age**	0.703 (0.699, 0.707)	0.893 (0.873, 0.914)	0.996 (0.975, 1.016)	0.006 (0.005, 0.006)	0.007 (0.006, 0.007)
**MLR_age**	0.710 (0.707, 0.714)	0.923 (0.901, 0.944)	0.996 (0.975, 1.017)	0.005 (0.004, 0.006)	0.008 (0.008, 0.009)

Supplementary file 12: zoom-in plots of performance metrics for dynamic models

Figure S6: Zoom-in plots of performance metrics for dynamic models

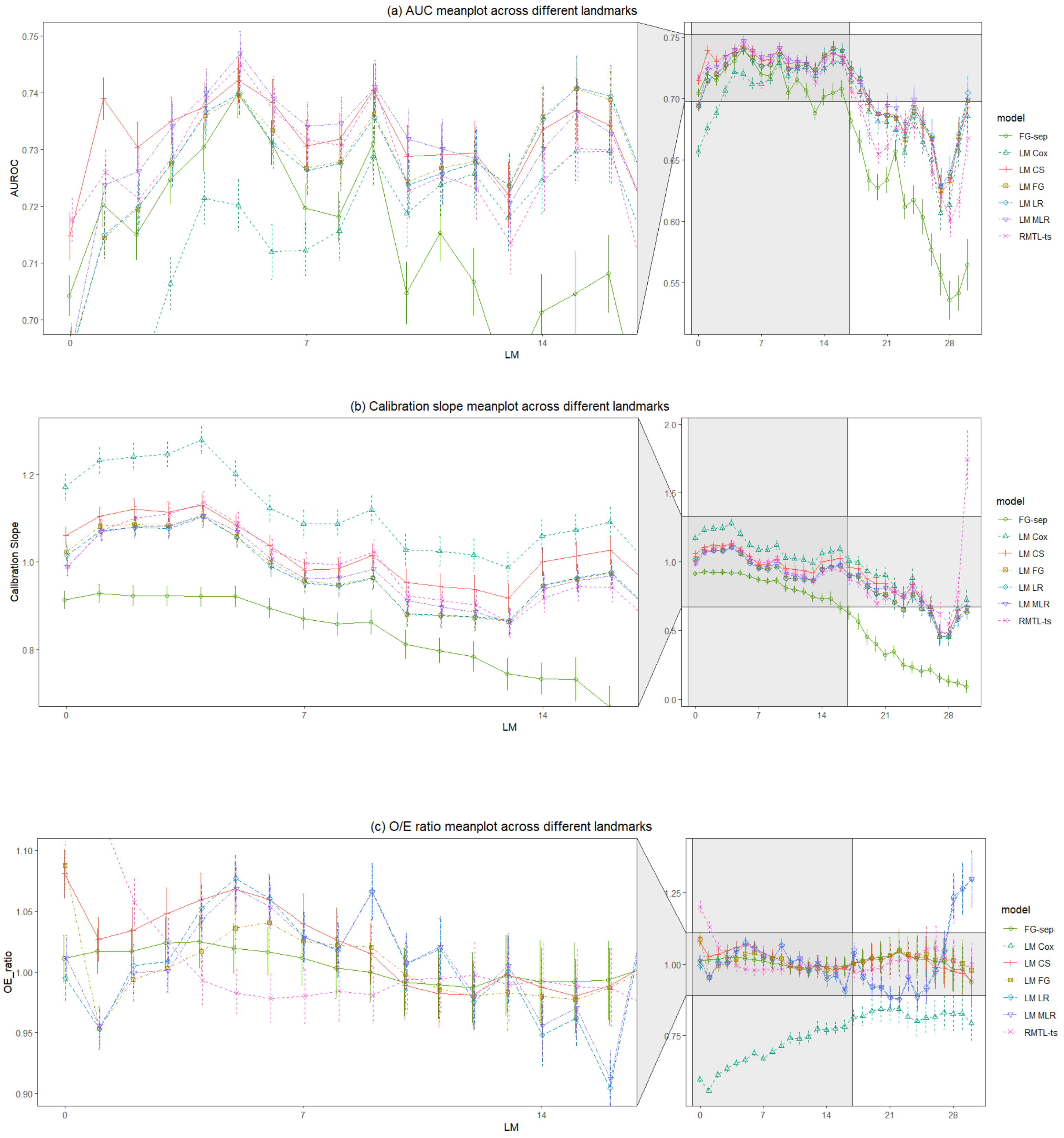

(d) ECI meanplot across different landmarks

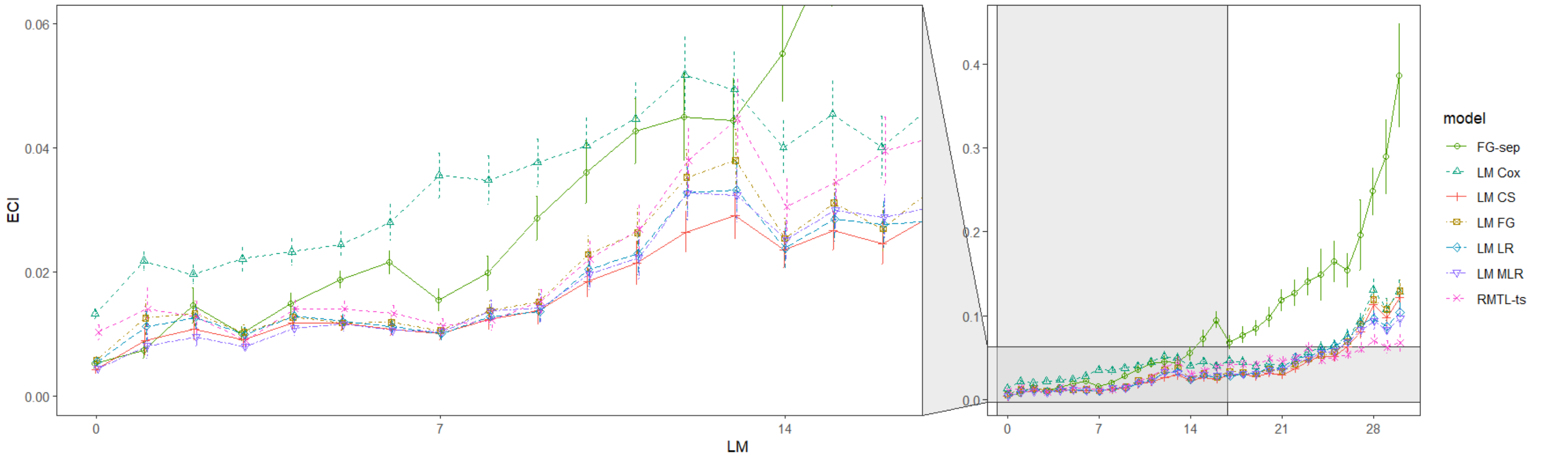

(e) Scaled BS meanplot across different landmarks

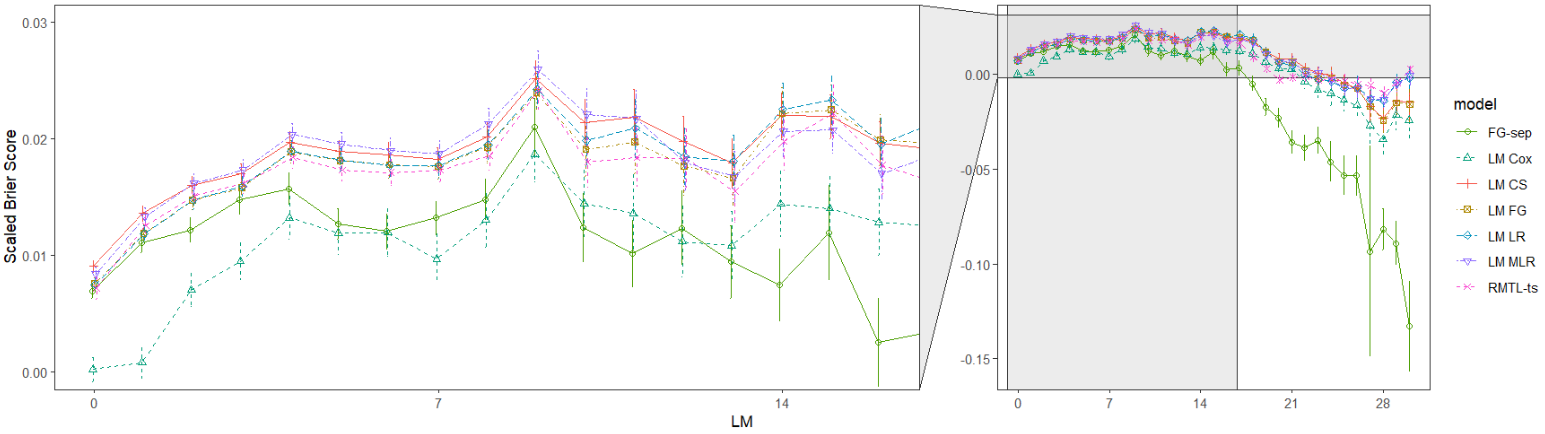

Supplementary file 13: separate fine-gray landmark model

We fitted separate fine-gray models to our landmark super datasets. It shall be noticed that convergence started failure since landmark day 14 for separate Fine-Gray landmark model. The number of convergence failures is shown in Table S10.

Table S10: number of convergence failure per landmark for separate Fine-Gray landmark model

LM	Nr of convergence failure (fg_model$crrFit$converged==FALSE)
30	99
29	99
28	100
27	100
26	100
25	98
24	100
23	100
22	100
21	100
20	17
19	16
18	12
17	12
16	12
15	7
14	1

Figure S7: comparison of performance metrics of dynamic models with inclusion of age

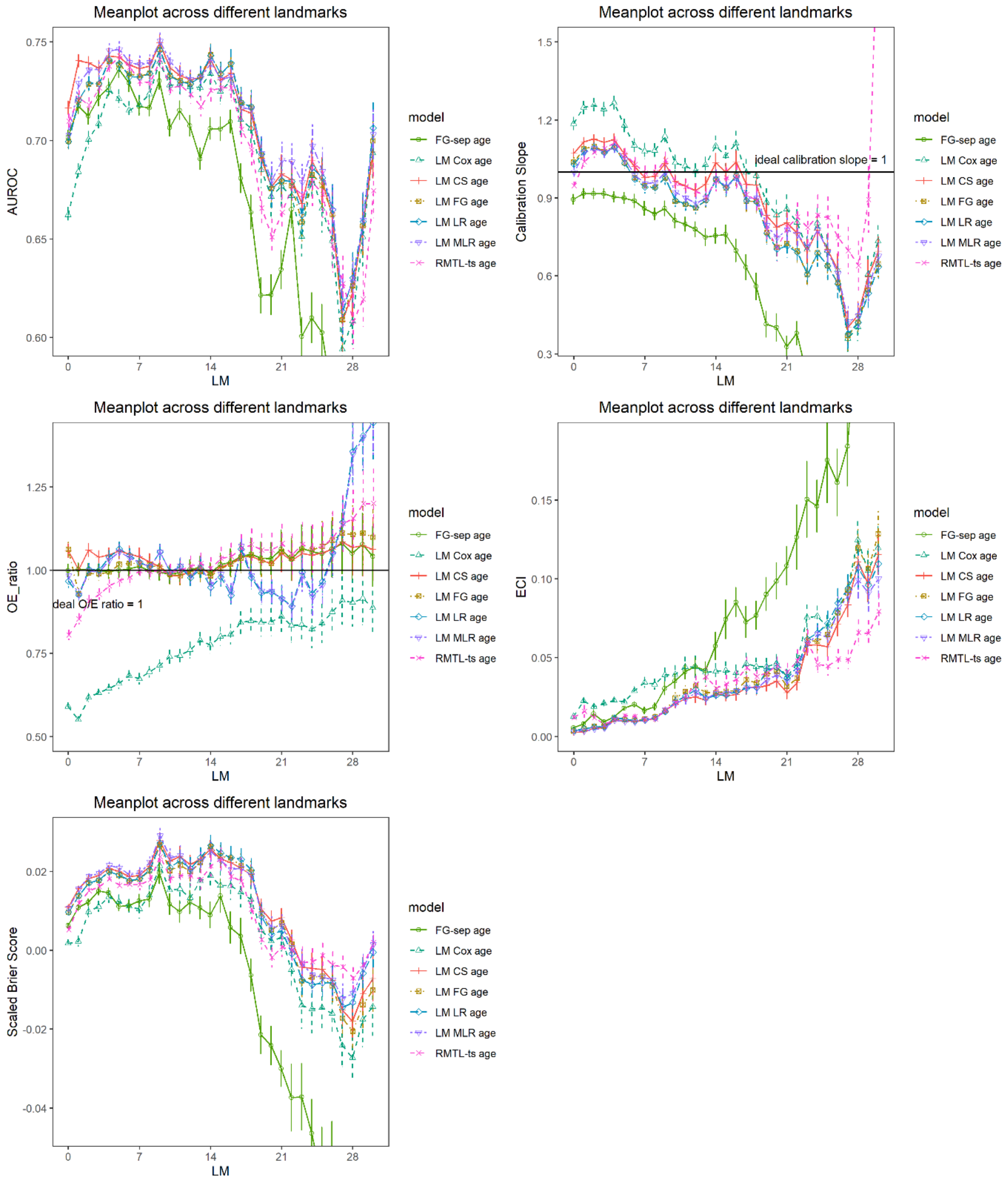

Supplementary file 14: distribution of predicted risks of dynamic models per landmark

Figure S8 distribution plot of predicted risks of dynamic models over landmark time points

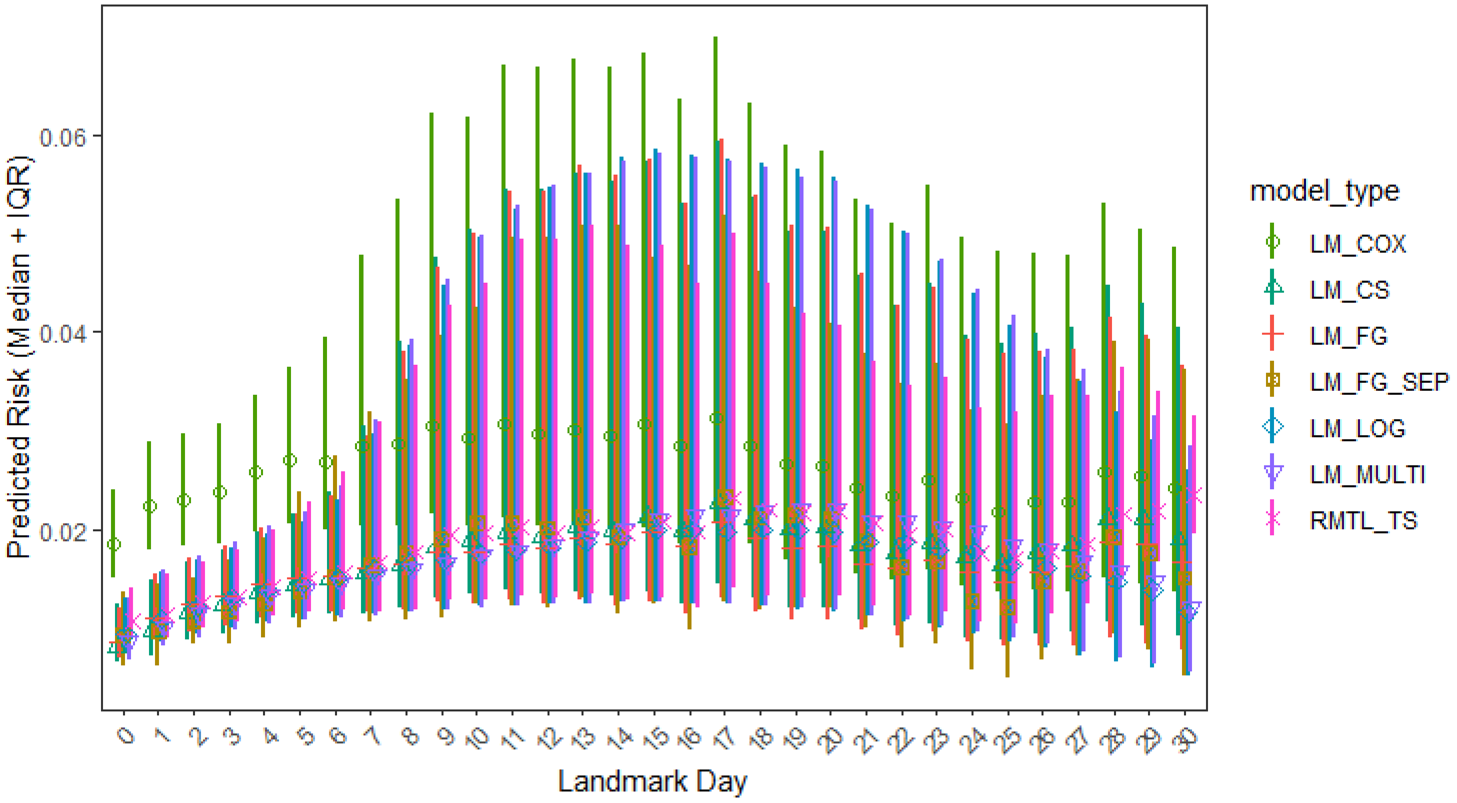

Supplementary file 15: calibration plots of the landmark cause-specific model per landmark

Figure S9: calibration plots of the landmark cause-specific model for 100 train-test splits per landmark

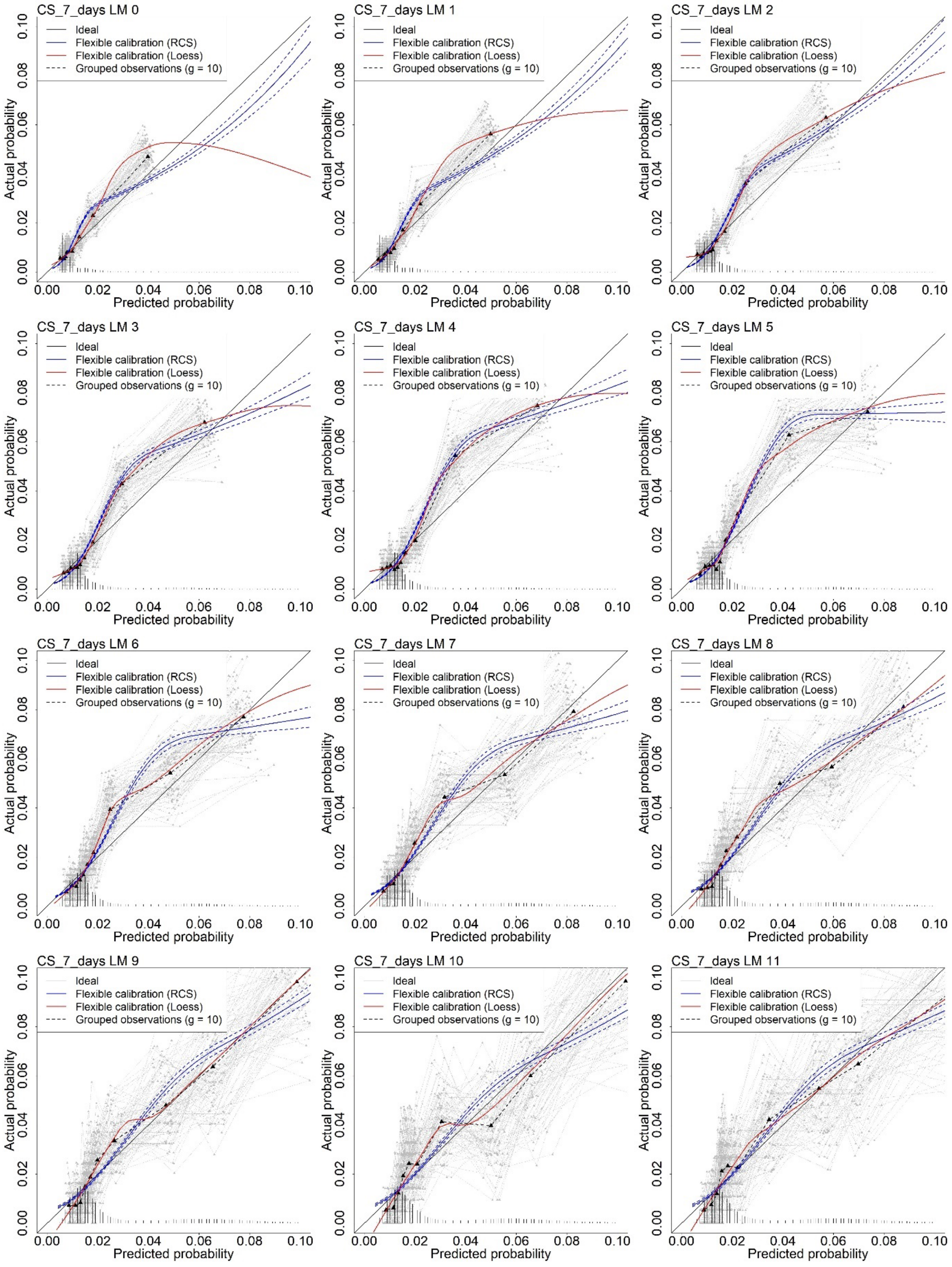

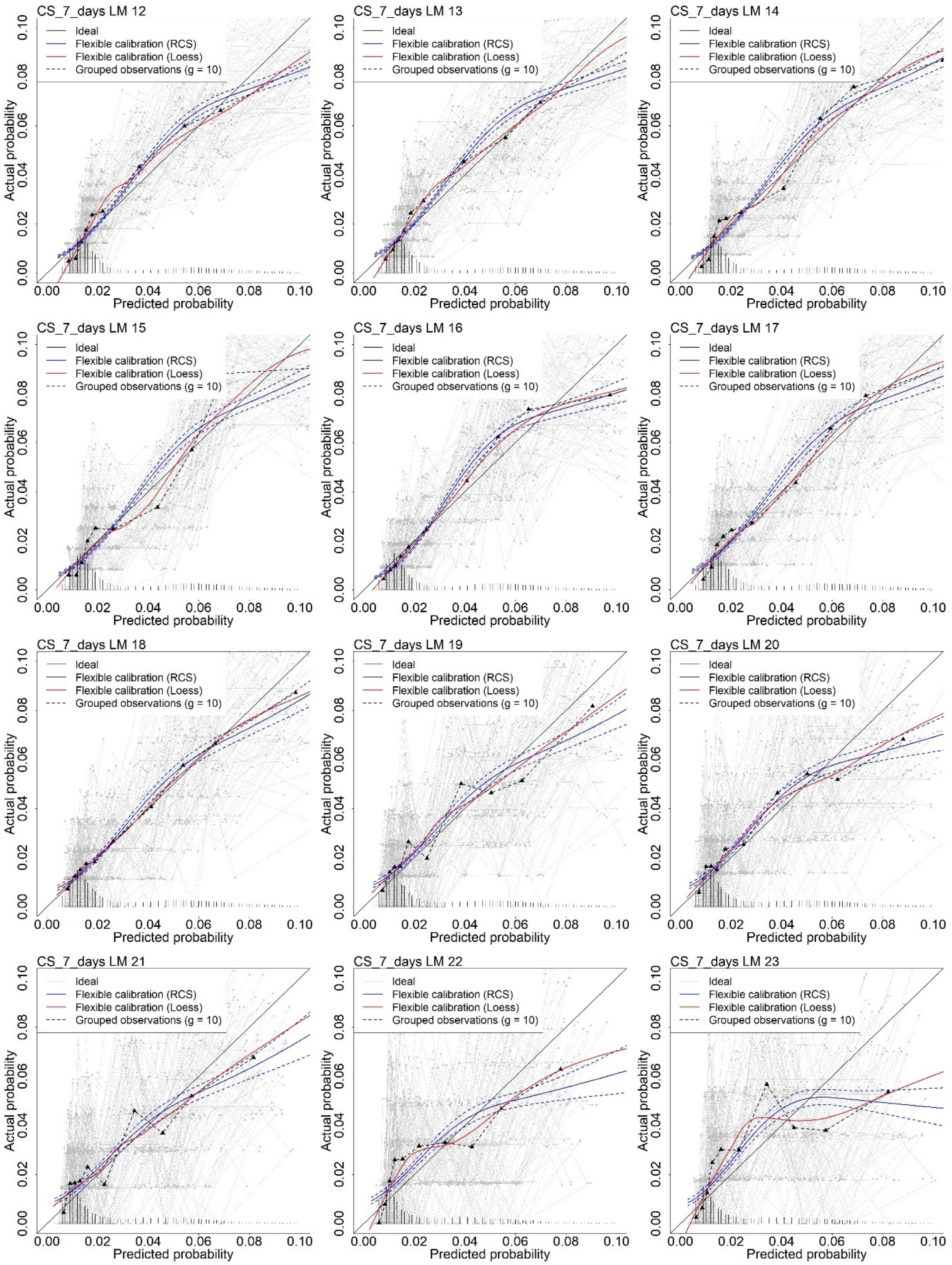
CS_7_days LM 12
CS_7_days LM 13
CS_7_days LM 14
CS_7_days LM 15
CS_7_days LM 16
CS_7_days LM 17
CS_7_days LM 18
CS_7_days LM 19
CS_7_days LM 20
CS_7_days LM 21
CS_7_days LM 22
CS_7_days LM 23
Ideal
Flexible calibration (RCS)
Flexible calibration (Loess)
Grouped observations (g = 10)
Actual probability
Predicted probability
0.00
0.02
0.04
0.06
0.08
0.10

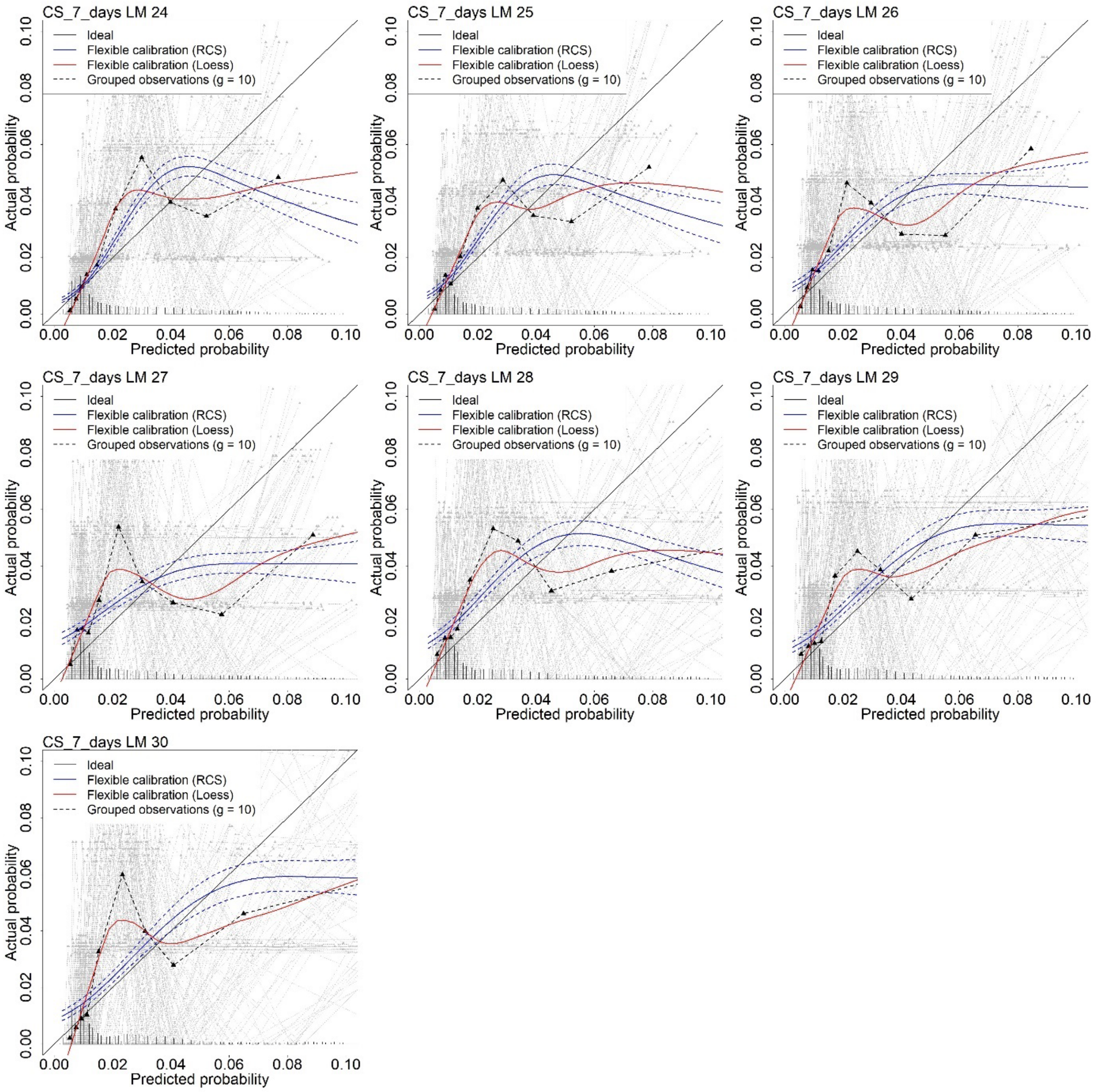

CS_7_days LM 24
CS_7_days LM 25
CS_7_days LM 26
CS_7_days LM 27
CS_7_days LM 28
CS_7_days LM 29
CS_7_days LM 30
Ideal
Flexible calibration (RCS)
Flexible calibration (Loess)
Grouped observations (g = 10)
Actual probability
Predicted probability
0.00
0.02
0.04
0.06
0.08
0.10

References

1. Centers for Disease Control and Prevention. Bloodstream Infection Event (Central Line-Associated Bloodstream Infection and Non-central Line Associated Bloodstream Infection) [Internet]. 2022 Jan. Available from: https://www.cdc.gov/nhsn/pdfs/pscmanual/4psc_clabscurrent.pdf.

2. Duysburgh, E. 2019. "Surveillance Bloedstroom Infecties in Belgische Ziekenhuizen - Protocol 2019." Brussel, België: Sciensano. https://www.sciensano.be/sites/default/files/bsi_surv_protocol_nl_april2019.pdf.

3. VAN HOUWELINGEN, H.C. (2007), Dynamic Prediction by Landmarking in Event History Analysis. Scandinavian Journal of Statistics, 34: 70-85. https://doi.org/10.1111/j.1467-9469.2006.00529.x.
4. Houwelingen, Hans, and Hein Putter. Dynamic Prediction In Clinical Survival Analysis. Boca Raton: CRC Press, 2012.
5. Steyerberg, Ewout. (2009). Clinical Prediction Models: A Practical Approach to Development, Validation, and Updating. 10.1007/978-0-387-77244-8.
6. Hashimoto EM, Ortega EMM, Cordeiro GM, Suzuki AK, Kattan MW. The multinomial logistic regression model for predicting the discharge status after liver transplantation: estimation and diagnostics analysis. J Appl Stat. 2019 Dec 24;47(12):2159-2177. doi: 10.1080/02664763.2019.1706725. PMID: 35706842; PMCID: PMC9041638.
7. Benichou J, Gail MH. Estimates of absolute cause-specific risk in cohort studies. Biometrics. 1990 Sep;46(3):813-26. PMID: 2242416.
8. Fine JP, Gray RJ. A proportional hazards model for the subdistribution of a competing risk. Journal of the American Statistical Association. 1999;446:496–509.
9. van Houwelingen HC, Putter H. Comparison of stopped Cox regression with direct methods such as pseudo-values and binomial regression. Lifetime Data Anal. 2015 Apr;21(2):180-96. doi: 10.1007/s10985-014-9299-3. Epub 2014 Aug 2. PMID: 25084763.
10. Anya H Fries, Eunji Choi, Julie T Wu, Justin H Lee, Victoria Y Ding, Robert J Huang, Su-Ying Liang, Heather A Wakelee, Lynne R Wilkens, Iona Cheng, Summer S Han, Software Application Profile: dynamicLM—a tool for performing dynamic risk prediction using a landmark supermodel for survival data under competing risks, International Journal of Epidemiology, 2023, https://doi.org/10.1093/ije/dyad122.
11. Ozenne, Brice & Sørensen, A.L. & Scheike, Thomas & Torp-Pedersen, Christian & Gerds, Thomas. (2017). riskRegression: Predicting the Risk of an Event using Cox Regression Models. R Journal. 9. 440-460. 10.32614/RJ-2017-062.
12. Nicolaie MA, van Houwelingen JC, de Witte TM, Putter H. Dynamic prediction by landmarking in competing risks. Stat Med. 2013 May 30;32(12):2031-47. doi: 10.1002/sim.5665. Epub 2012 Oct 22. PMID: 23086627.
13. Qing Liu, Gong Tang, Joseph P. Costantino, Chung-Chou H. Chang, Landmark Proportional Subdistribution Hazards Models for Dynamic Prediction of Cumulative Incidence Functions, Journal of the Royal Statistical Society Series C: Applied Statistics, Volume 69, Issue 5, November 2020, Pages 1145–1162, https://doi.org/10.1111/rssc.12433.
14. Geskus RB. Cause-specific cumulative incidence estimation and the fine and gray model under both left truncation and right censoring. Biometrics. 2011 Mar;67(1):39-49. doi: 10.1111/j.1541-0420.2010.01420.x. PMID: 20377575.
15. Therneau TM. A Package for Survival Analysis in R [Internet]. 2020. Available from: https://CRAN.R-project.org/package=survival.
16. Ngwa, J.S., Cabral, H.J., Cheng, D.M. et al. A comparison of time dependent Cox regression, pooled logistic regression and cross sectional pooling with simulations and an application to

the Framingham Heart Study. BMC Med Res Methodol 16, 148 (2016). https://doi.org/10.1186/s12874-016-0248-6.
17. Cao H, Zhou J, Schwarz E. RMTL: an R library for multi-task learning. Bioinformatics. 2019 May 15;35(10):1797-1798. doi: 10.1093/bioinformatics/bty831. PMID: 30256897.
18. Riley RD, Ensor J, Snell KIE, Harrell FE Jr, Martin GP, Reitsma JB, Moons KGM, Collins G, van Smeden M. Calculating the sample size required for developing a clinical prediction model. BMJ. 2020 Mar 18;368:m441. doi: 10.1136/bmj.m441. PMID: 32188600.
19. Gao S, Albu E, Tuand K, Cossey V, Rademakers F, Van Calster B, Wynants L. Systematic review finds risk of bias and applicability concerns for models predicting central line-associated bloodstream infection. J Clin Epidemiol. 2023 Sep;161:127-139. doi: 10.1016/j.jclinepi.2023.07.019. Epub 2023 Aug 2. PMID: 37536503.
